# Sound-evoked adenosine release in cooperation with neuromodulatory circuits permits auditory cortical plasticity and perceptual learning

**DOI:** 10.1016/j.celrep.2024.113758

**Published:** 2024-02-13

**Authors:** Ildar T. Bayazitov, Brett J.W. Teubner, Feng Feng, Zhaofa Wu, Yulong Li, Jay A. Blundon, Stanislav S. Zakharenko

**Affiliations:** 1 Division of Neural Circuits and Behavior, Department of Developmental Neurobiology, St. Jude Children’s Research Hospital, Memphis, TN 38105, USA; 2 School of Life Sciences, Peking University, Beijing 100871, China; 3 Lead contact

## Abstract

Meaningful auditory memories are formed in adults when acoustic information is delivered to the auditory cortex during heightened states of attention, vigilance, or alertness, as mediated by neuromodulatory circuits. Here, we identify that, in awake mice, acoustic stimulation triggers auditory thalamocortical projections to release adenosine, which prevents cortical plasticity (i.e., selective expansion of neural representation of behaviorally relevant acoustic stimuli) and perceptual learning (i.e., experience-dependent improvement in frequency discrimination ability). This sound-evoked adenosine release (SEAR) becomes reduced within seconds when acoustic stimuli are tightly paired with the activation of neuromodulatory (cholinergic or dopaminergic) circuits or periods of attentive wakefulness. If thalamic adenosine production is enhanced, then SEAR elevates further, the neuromodulatory circuits are unable to sufficiently reduce SEAR, and associative cortical plasticity and perceptual learning are blocked. This suggests that transient low-adenosine periods triggered by neuromodulatory circuits permit associative cortical plasticity and auditory perceptual learning in adults to occur.

## In brief

Cortical plasticity occurs in adults when sensory circuits and neuromodulatory circuits are co-activated. Bayazitov et al. report that this plasticity is prevented when acoustic stimulation triggers thalamic adenosine release. Neuromodulatory (cholinergic or dopaminergic) inputs transiently curb this adenosine release to enable auditory cortical plasticity and perceptual learning to occur.

## Graphical Abstract

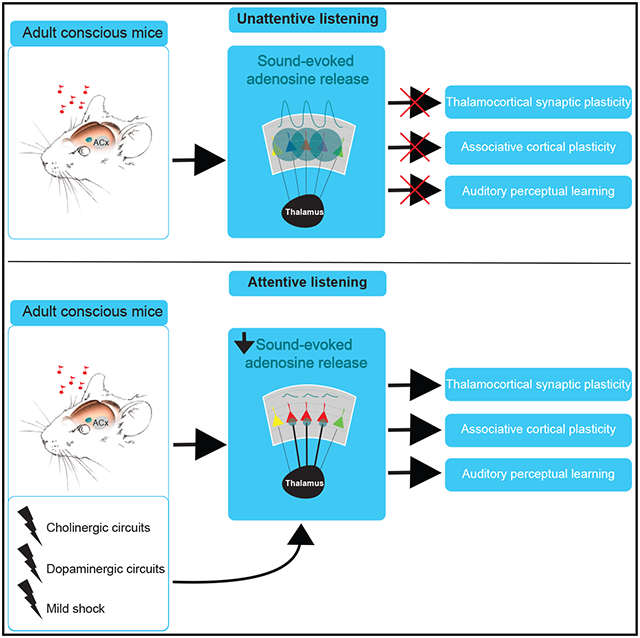

## INTRODUCTION

Perceptual learning produces long-lasting changes (perceptual memory) to an organism’s sensory systems (visual, tactile, olfactory, gustatory, and auditory) that improve its ability to survive and interact with the environment.^1,2^ Auditory perceptual learning (or auditory learning) improves the detection and segregation of behaviorally important sounds, which is essential for communication and, in humans, for language and music processing.^3-6^

Auditory learning is a manifestation of neural plasticity—defined as experience-dependent changes in neural circuits––in the auditory cortex (ACx).^7-12^ Neural circuits in sensory cortices are plastic; i.e., their properties change as new environmental information is integrated based on its behavioral value throughout life.^13-22^ However, they use different strategies over the lifespan as the ability of the external environment alone to drive plasticity in the ACx sharply declines with age.

During the early critical period (in rodents, post-natal day 11 [P11]–P15), passive exposure to an acoustic stimulus can induce auditory cortical plasticity in the form of persistent expansion of neural representation in the ACx specific to that stimulus frequency.^23-25^ After the early critical period ends, the ability of passive sound exposure alone to induce ACx plasticity is severely diminished. In adults, ACx plasticity is induced during attentive wakefulness^9,10,26-29^ or by pairing acoustic stimuli with activation of neuromodulatory circuits, such as the cholinergic circuits emanating from the nucleus basalis (NB),^7,30-37^ dopaminergic circuits emanating from the ventral tegmental area (VTA),^38,39^ or norepinephrinergic circuits emanating from the locus coeruleus.^40-42^ These neuromodulators, which are thought to heighten attention, vigilance, alertness, and active task engagement, are released during various salient stimuli and thus may filter incoming sensory information based on its behavioral importance.^31,43-51^ This form of cortical plasticity is associative in nature; i.e., a conditioned stimulus (CS; such as sound) is paired with an unconditioned stimulus (US; such as mild shock), where the latter causes neuromodulator release^50,52,53^ to establish a persistent, selective potentiation of neural representation of the CS frequency in the ACx.^19,54^

Alternatively, ACx plasticity in adult mice can be induced by passive exposure to an acoustic stimulus when adenosine signaling in the thalamus is experimentally reduced.^55^ Adenosine, acting through presynaptic A1 receptors (A_1_Rs), is a negative regulator of neurotransmitter release, mainly at glutamatergic synapses.^56-58^ Adenosine is released extracellularly in an activity-dependent manner,^56,57^ acts in seconds over a diffuse area,^59^ and originates in part from ecto-5'-nucleotidase (NT5E or CD73) catalyzing the last step of ATP metabolism.^60,61^ In the thalamus and ACx, adenosine levels are high in adults and low in pups, contingent on NT5E activity.^55^ Pharmacological inhibition or genetic reduction of adenosine production by NT5E or that of A_1_R signaling in the thalamocortical (TC) projections to the ACx produces a chronic “low-adenosine condition” that emulates the juvenile early critical period. Thus, under the chronic low-adenosine condition, passive exposure to an acoustic stimulus alone induces long-lasting, stimulus-specific cortical plasticity in the ACx and hones auditory acuity in adult mice.^55^

The *in vivo* findings described above are supported by observations in brain slices. Specifically, long-term potentiation (LTP) and long-term depression (LTD) at TC synapses, both of which are forms of long-term synaptic plasticity that are believed to underlie cortical plasticity in sensory cortices,^58,62^ sharply decline (unlike other glutamatergic synapses in sensory cortices) after the early critical period ends.^63-65^ TC LTP and LTD do not disappear in mature animals, but rather they become gated via adenosine-mediated presynaptic mechanisms. Indeed, inhibition of NT5E or A_1_Rs restores TC LTP/LTD in the adult ACx to their juvenile levels.^66,67^ Furthermore, the negative effect of adenosine on glutamate release and TC LTP/LTD in the ACx can be relieved by activation of cholinergic projections through activation of muscarinic receptors.^66,67^

Despite parallels between the effects of neuromodulatory projections and those of adenosine machinery on adult ACx plasticity, how these systems work together *in vivo* to produce ACx plasticity and enable auditory learning in adults is unclear. Here we report that acoustic stimuli evoke the release of adenosine in the ACx of awake adult wild-type (WT) mice, and this sound-evoked adenosine release (SEAR) is dampened by neuromodulatory circuits to produce a transient juvenile-like state that permits both ACx plasticity and auditory learning.

## RESULTS

### SEAR in the ACx

We measured the adenosine levels during sound stimulation in awake mice by using fast-scan cyclic voltammetry (FSCV), which detects extracellular adenosine with sub-second temporal resolution.^68,69^ We also used two-photon imaging of the genetically encoded fluorescent G-protein-coupled receptor (GPCR) sensor GRAB_Ado_ (GPCR-activation-based adenosine [Ado]), which detects spatiotemporal dynamics of extracellular adenosine.^70,71^ We measured cyclic voltammograms *in vitro* at 1.4 V, which is highly selective for adenosine oxidation,^69^ to ensure specificity. FSCV was sensitive to adenosine but not to ATP or its metabolites (ADP and AMP), the nucleoside guanosine, the nucleotides inosine and guanine, or the neuromodulators dopamine and acetylcholine (Figure S1).

**Figure 1.**
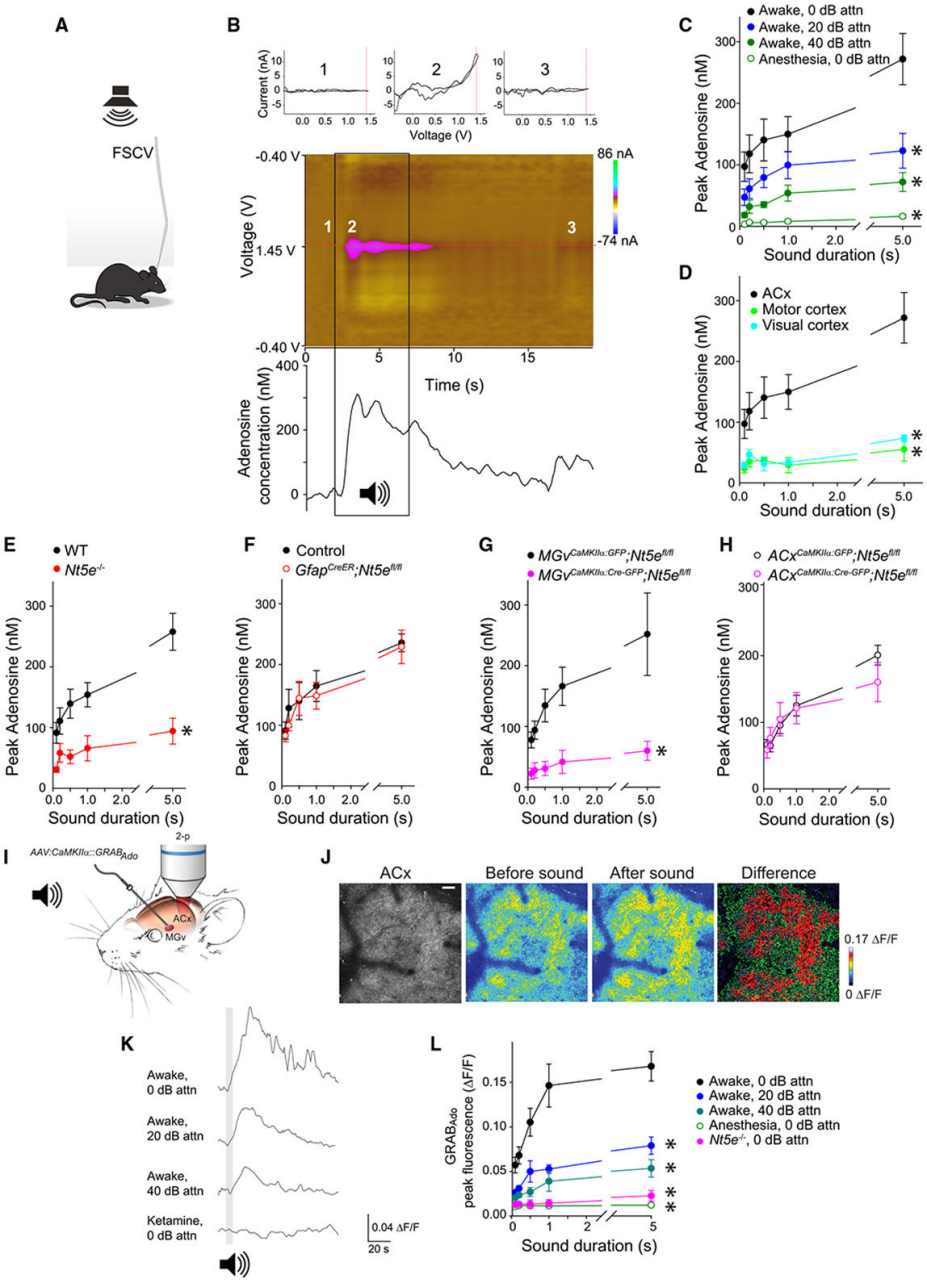
SEAR in the ACx of adult mice (A) Schematic of fast-scan cyclic voltammetry (FSCV) in awake mice. A carbon fiber microelectrode is implanted into the auditory cortex (ACx), and adenosine levels are recorded in response to sounds (denoted as a speaker). (B) Top: representative *in vivo* FSCV of adenosine in the mouse ACx. The applied potential was scanned from −0.4 to 1.5 V and back at 400 V/s every 100 ms. Shown are representative background-subtracted cyclic voltammograms before (1), during (2), and after (3) delivery of 5-s broadband noise. A red line at 1.4 V indicates the oxidation peak of adenosine. Center: false-color plot of a sound-evoked adenosine transient in response to broadband noise (denoted as a speaker) with a red line marking the primary adenosine oxidation voltage at 1.4 V. Numbers represent epochs before (1), during (2), and after (3) delivery of a 5-s broadband noise. Bottom: current vs. time trace of an adenosine transient in response to a 5-s broadband noise (black box). (C) Mean peak amplitude of SEAR as a function of broadband noise duration and intensity (attenuation [attn]) in awake mice and anesthetized mice; 2-way repeated-measures (RM) ANOVA, *F*_*3,4*_ = 7.4, *p < 0.001; sound durations, *p < 0.001; anesthesia and attn, *p < 0.001; 5 mice). (D) SEAR is detected in the ACx but not in the motor or visual cortices (2-way RM ANOVA, *F*_*2,4*_ = 4.6, *p < 0.001; ACx, 5 mice; motor and visual cortices, 3 mice). (E) Average peak SEAR in the ACx measured in WT (n = 7) and *Nt5e*^*−/−*^ (n = 6) mice (2-way RM ANOVA, *F*_*1,4*_ = 7.3, *p < 0.001; genotype, *p = 0.004). (F) Conditional deletion of *Nt5e* in astroglia does not affect SEAR in the ACx (2-way RM ANOVA, *F*_*1,4*_ = 0.21, p = 0.930; 3 *Gfap^CreER^;Nt5e^fl/fl^* mice, 3 *Gfap^CreER^;Nt5e^+/+^* mice). (G and H) Conditional deletion of Nt5e in excitatory neurons in the auditory thalamus (MGv) (G: 2-way RM ANOVA, *F*_*1,4*_ = 4.392, *p = 0.007; genotype, *p = 0.02; 5 *MGv^CaMKIIa:GFP^;Nt5^fl/fl^* mice; 4 *MGv^CaMKIIa:Cre-GFP^;Nt5^fl/fl^* mice) but not in the ACx (H: 2-way RM ANOVA, *F*_*1,4*_ = 0.07, p = 0.797; 4 *ACx^CaMKIIa:GFP^;Nt5^fl/fl^* mice, 5 *ACx^CaMKIIa:Cre-GFP^;Nt5^fl/fl^* mice) reduced SEAR in the ACx. (I) Schematic for measuring SEAR in the ACx by two-photon (2-p) imaging of GRAB_Ado_ fluorescence in awake head-constrained mice. *AAV-hSyn:GRAB_Ado-1M_* was injected into the MGv several weeks before the imaging experiments were done. (J) Representative phase-contrast GRAB_Ado_ fluorescence images, before and after broadband noise, and their differences in the ACx of awake mice. Scale bar, 50 μm. (K) GRAB_Ado_ fluorescence measured in the L3/4 neurons of the ACx in response to broadband noise in awake mice and anesthetized mice. (L) Average peak GRAB_Ado_ fluorescence as a function of duration of broadband noise delivered at different intensities (attns) in awake mice (n = 2–12 animals), mice anesthetized with ketamine or phenobarbital (n = 6 animals), and awake *Nt5e*^*−/−*^ mice (n = 3 animals) (2-way RM ANOVA, *F*_*4,4*_ = 2.7, *p = 0.003; conditions, *p < 0.001). Holm-Sidak post hoc: control (awake 0-dB attn) vs. anesthesia, *p < 0.001; control vs. *Nt5e*^*−/−*^ mice, *p < 0.001; control vs. 20-dB attn, *p = 0.005; control vs. 40-dB attn, *p = 0.001. Averaged data are presented as the mean ± SEM.

In FSCV experiments *in vivo*, we delivered broadband noise or pure tones at multiple frequencies and intensities to tethered but mobile mice with a microelectrode implanted in their ACx (Figure 1A). Cyclic voltammograms measured at 1.4 V detected an increased adenosine level in the ACx evoked by broadband noise (white noise, 0–50 kHz) (Figure 1B). SEAR was proportional to the duration and intensity of the acoustic stimuli (Figure 1C) and concurrent with the onset of the acoustic stimulus (latency 0.44 ± 0.038 s from the onset, latency jitter 0.17 ± 0.023 s, 5 recording sites, 3 mice). SEAR rise time ranged from 1.07 ± 0.25 s (100-ms sound duration) to 2.55 ± 0.54 s (5-s sound duration), and decay time ranged from 2.41 ± 0.55 s (100-ms sound duration) to 6.78 ± 1.7 s (5-s sound duration, 6 mice). We observed SEAR in the ACx of awake mice but not in that of anesthetized mice (Figures 1C andS2). SEAR in the ACx was not indiscriminate, but rather it was frequency dependent at individual cortical locations (Figure S3). SEAR was specific to the ACx and not detected in the motor or visual cortices (Figure 1D).

Using FSCV, we sought to identify the mechanisms and origin of SEAR in the ACx. Most extracellular adenosine production is thought to be controlled by NT5E,^60,61^ and SEAR was more than 2.5-fold lower (p = 0.004) in *Nt5e*^*−/−*^ mice than in WT littermates (Figure 1E). To determine the cellular source of SEAR, we engineered a mouse with a floxed *Nt5e* allele for cell-type-specific knockout (Figure S4) and conditionally deleted *Nt5e* from 3 candidate sources: astroglia, thalamic excitatory neurons, and cortical excitatory neurons. We ruled out astroglia as the source because SEAR in the ACx was unaffected in mice with inducible deletion of *Nt5e* in astrocytes. Tamoxifen-treated *Gfap^CreER^;Nt5e^fl/fl^* mice expressing cre recombinase (Cre) under control of the *GFAP* promoter had normal SEAR in the ACx compared with control tamoxifen-treated *Gfap^CreER^;Nt5e^+/+^* mice (Figure 1F). However, deletion of *Nt5e* in the thalamic excitatory neurons reduced SEAR in the ACx more than 3-fold (Figure 1G). SEAR in the ACx was significantly (p = 0.02) reduced in *Nt5e*^*fl/fl*^ mice injected with the recombinant adeno-associated virus (AAV) encoding Cre under control of the excitatory neuron-specific promoter *CaMKIIα* (*AAV-CaMKIIα:Cre-GFP*) into the auditory thalamus (i.e., the ipsilateral ventral portion of the medial geniculate nucleus [MGv]) (*MGv^CaMKIIα:Cre-GFP^;Nt5^fl/fl^* mice) compared with control *Nt5e*^*fl/fl*^ mice injected with *AAV-CaMKIIα:GFP* into the MGv (*MGv^CaMKIIα:GFP^;Nt5e^fl/fl^* mice) (Figure 1G). In comparison, SEAR in the ACx was not affected when we deleted *Nt5e* from excitatory neurons in the ACx. *ACx^CaMKIIα:Cre-GFP^;Nt5e^fl/fl^* and control *ACx^CaMKIIα:GFP^;Nt5e^fl/fl^* mice had indistinguishable SEAR (p = 0.797) measured in the AAV-infected area of the ACx (Figure 1H). Because *Nt5e* deletion in the excitatory thalamic neurons impaired SEAR in the ACx, we concluded that the major source of SEAR is glutamatergic TC projections.

We further confirmed TC-mediated SEAR in the ACx by imaging GRAB_Ado_. We injected *AAV-hSyn:GRAB*_*Ado-1M*_ into the MGv (Figure 1I) and a few weeks later measured robust, time-locked changes in GRAB_Ado_ fluorescence in the ACx in response to broadband noise (Figures 1J and 1K). GRAB_Ado-1M_ responds to adenosine but not to ATP.^71^ We confirmed GRAB_Ado-1M_ sensitivity to adenosine but not to adenine or inosine (Figures S5A and S5B). Furthermore, experiments in brain slices showed a strong correlation (*r^2^* = 0.984, p = 0.008) between FSCV and GRAB_Ado-1M_ responses to adenosine (Figure S5C). Together, these complementary methods confirmed TC SEAR *in vivo*.

The amplitude of the TC SEAR measured by the GRAB_Ado_ approach was proportional to the intensity and duration of the acoustic stimuli (Figures 1K and 1L). SEAR measured by GRAB_Ado_ was slower than that measured by FSCV, most likely due to the slower kinetics of GRAB_Ado_.^70^ SEAR rise time in response to 5-s broadband noise ranged from 2.5 ± 0.8 s (40-dB sound pressure level [SPL] attenuation) to 5.4 ± 2.2 s (no attenuation), and SEAR decay time ranged from 3.6 ± 1.3 s (40-dB SPL attenuation, 5 mice) to 10.3 ± 3.6 s (no attenuation, 10 mice).

Like FSCV, the GRAB_Ado_ approach determined that SEAR was blocked by general anesthesia (Figures 1K, 1L, and S5E-S5G) and in *Nt5e*^*−/−*^ mice (Figure 1L).

### Neuromodulatory circuits transiently decrease SEAR in an associative manner

**Figure 2.**
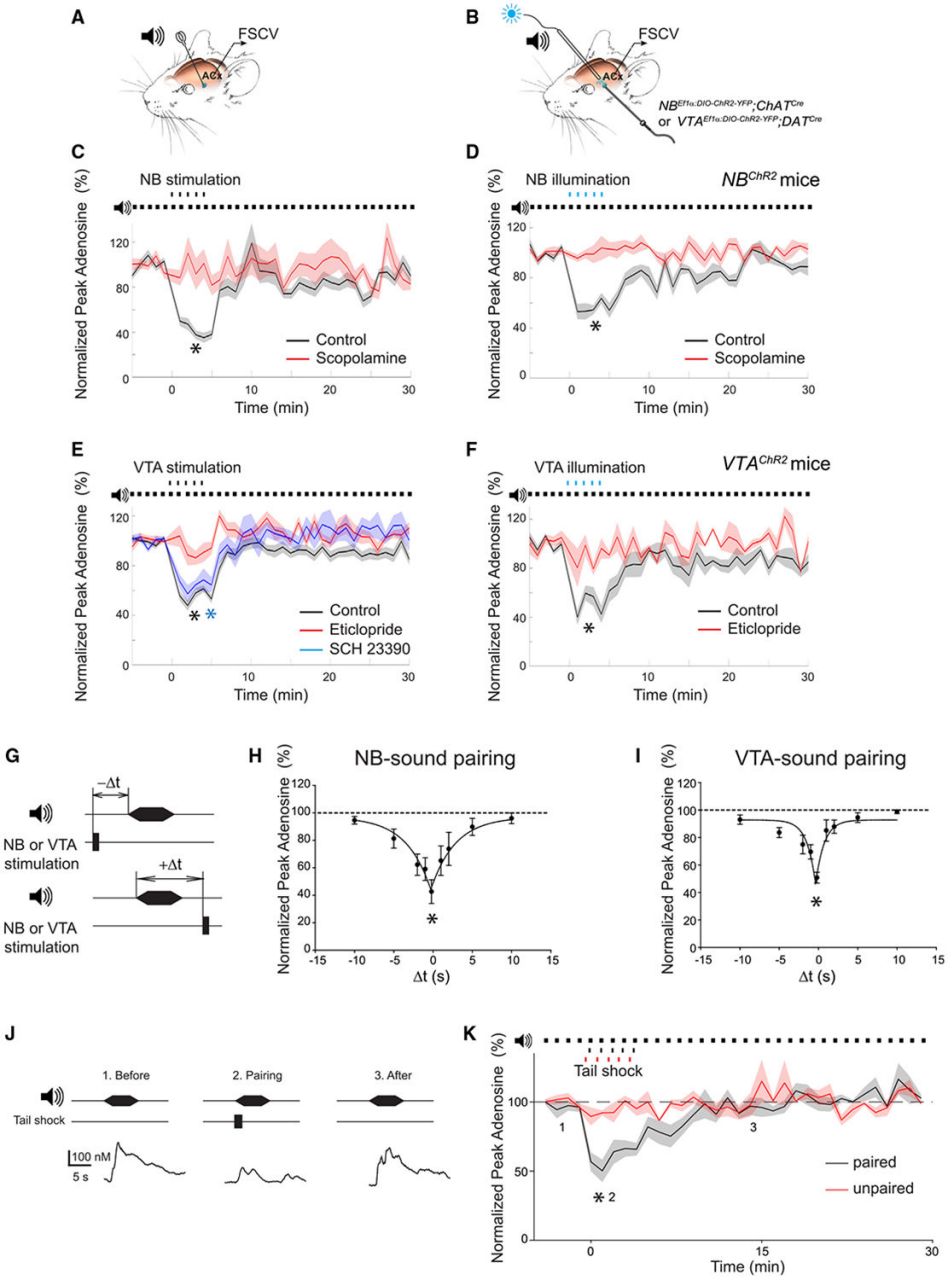
Aversive stimuli or activation of neuromodulatory projections dynamically reduce SEAR in the ACx of adult mice (A and B) Schematics of electrical (A) or optogenetic (B) activation of the NB or VTA cell bodies. A bipolar stimulating electrode was inserted into the NB or VTA of WT mice (A), and an optical fiber was inserted into the ChR2-expressing NB or VTA of *ChAT*^*Cre*^ or *DAT*^*Cre*^ mice, respectively (B). (C and D) Mean normalized peak SEAR in the ACx measured in response to broadband noise (5 s) before, during, and after pairing with NB electrical stimulation (C, 5 black dots) or NB optical stimulation (D, 5 blue dots) in control (electrical: paired t test *t*_*18*_ = 16.1, 2-tailed *p < 0.001 relative to baseline, 14 mice; optical: paired t test, *t*_*6*_ = 11.1, 2-tailed *p < 0.001 relative to baseline, 6 mice) or in scopolamine-treated mice (electrical: paired t test, *t*_*12*_ = 0.56, 2-tailed p = 0.59 relative to baseline, 10 mice; optical: paired t test, *t*_*5*_ = 0.52, 2-tailed p = 0.63 relative to baseline, 4 mice). In optogenetic experiments (D), the NBs of *ChAT*^*Cre*^ mice were injected with *AAV-Ef1a-DIO-hChR2(E123T/T159C)-EYFP* (*NB*^*ChR2*^ mice). (E and F) Mean normalized peak SEAR in the ACx as a function of time measured before, during, and after pairing broadband noise with electrical (E, 5 black dots) or optical (F, 5 blue dots) stimulation of the VTA in control (electrical: paired t test, *t*_*23*_ = 19.1, 2-tailed *p < 0.001 relative to baseline, 13 mice; optical: paired t test, *t*_*6*_ = 7.4, 2-tailed *p < 0.001 relative to baseline, 5 mice), eticlopride-treated (electrical: paired t test, *t*_*10*_ = 1.4, 2-tailed p = 0.19 relative to baseline, 7 mice; optical: paired t test, *t*_*4*_ = 1.3, 2-tailed p = 0.29 relative to baseline, 3 mice), or SCH 23390-treated mice (electrical: Wilcoxon signed-rank test, *W*_*8*_ = −36, *p = 0.008 relative to baseline, 6 mice). In optogenetic experiments (F), *DAT*^*Cre*^ mice were injected into the VTA with *AAV-Ef1a-DIO-hChR2(E123T/T159C)-EYFP* (*VTA^ChR2^* mice). (G) Schematics of pairing an acoustic stimulus with electrical stimulation of the NB or VTA. (H and I) Normalized mean peak SEAR in ACx as a function of time delay between acoustic stimulus and NB (H) or VTA (I) stimulation in the respective pairing protocols (1-sample t test, Dt = 0.2 s, NB: *t*_*4*_ = −6.6, 2-tailed *p = 0.003; VTA: *t*_*4*_ = −12.4, 2-tailed *p < 0.001; Dt = −10 s and Dt = 10 s, p > 0.05, 5 mice each). Global fit curves (pseudo-Voigt approximation with 5 parameters) are also shown. (J) Representative SEAR in the ACx before, during, and after pairing broadband noise (5 s) with tail shock. (K) Mean normalized peak SEAR as a function of time before, during, and after paired (5 black dots, 300–400 ms between the tail shock and sound) or unpaired (5 red dots, 20 s between the tail shock and sound) delivery of tail shocks (5 dots) and broadband noise (5 s). Paired stimulation: paired t test, *t*_*7*_ = 9.554, 2-tailed *p = 0.0003 relative to baseline, 8 mice. Unpaired stimulation: paired t test, *t*_*2*_ = 3.9, 2-tailed p = 0.06 relative to baseline, 3 mice. Dashed line, baseline. Data are presented as the mean ± SEM.

We next tested whether neuromodulatory (cholinergic or dopaminergic) projections regulate SEAR in the ACx. The main subcortical source of cholinergic innervation to cortical areas and a major nucleus responsible for cortical plasticity is the NB.^17,19,31,32,49,53,72-74^ After establishing the baseline at which SEAR was evoked repeatedly by broadband noise, we paired broadband noise with NB stimulation (NB-sound pairing) by either electrical (Figure 2A) or optogenetic (Figure 2B) means. NB-sound pairing reduced SEAR in the ACx in an activity-dependent manner. In electrical experiments, NB-sound pairing reduced SEAR by 58.5% ± 6.4%, relative to baseline (p < 0.001) (Figure 2C). Similarly, SEAR was transiently reduced by 44.3% ± 4.4% relative to baseline (p < 0.001) when we paired illumination of channelrhodopsin (ChR2)-expressing NB neurons with broadband noise in *ChAT*^*Cre*^ mice injected with *AAV-Ef1α-DIO-hChR2(E123T/T159C)-EYFP* into the basal forebrain (*NB*^*ChR2*^ mice) (Figure 2D). SEAR reduction induced by NB-sound pairing depended on synaptic cholinergic mechanisms, as it was inhibited by the muscarinic receptor antagonist scopolamine (3 mg/kg) delivered by intraperitoneal (i.p.) injection (Figures 2C and 2D). In the presence of scopolamine, NB-sound pairing failed to reduce SEAR by either electrical (5.5% ± 11.1%, p = 0.59, relative to baseline) or optogenetic (−1.5% ± 7.9%, p = 0.63, relative to baseline) approaches. This result is consistent with muscarinic receptors being required for ACx plasticity induced by NB-sound pairing^19,75^ and for TC synaptic plasticity in adults.^58,66,67^

Pairing acoustic stimuli with stimulation of dopaminergic neurons also reduced SEAR in the ACx (Figures 2E and 2F). Dopaminergic projections in the cortex mostly originate in the VTA,^76^ are activated by salient or aversive stimuli,^48^ and if paired with sounds, induce ACx plasticity in adults.^38^ When we paired broadband noise with electrical stimulation of the VTA (VTA-sound pairing), SEAR in the ACx was reduced by 44.8% ± 5.1% (p < 0.001) (Figure 2E). In a similar fashion, VTA-sound pairing induced optogenetically in *DAT*^*cre*^ mice expressing ChR2 in VTA neurons (injected with *AAV-Ef1a-DIO-hChR2(E123T/T159C)-EYFP* into the VTA; *VTA*^*ChR2*^ mice) reduced SEAR in the ACx by 47.9% ± 10.1% relative to baseline (p < 0.001) (Figure 2F). This SEAR reduction depended on dopaminergic synaptic transmission. Eticlopride (0.5 mg/kg, i.p. injection), a D2/D3 dopamine receptor antagonist, blocked SEAR reduction in the ACx, whether it was induced by electrical VTA-sound pairing (7.3% ± 6.9%, p = 0.19 relative to baseline) or optogenetic VTA-sound pairing (11.0% ± 8.8%, p = 0.29 relative to baseline) (Figures 2E and 2F). In contrast, SCH 23390 (0.5 mg/kg, i.p. injection), a selective blocker of D1 dopamine receptors, did not prevent SEAR reduction in the ACx (35.6% ± 4.3%, p = 0.008, relative to baseline), which was induced by electrical VTA-sound pairing (Figure 2E).

Both NB- and VTA-sound pairing protocols transiently reduced SEAR, dependent on the delay between the sound presentation and the stimulation of NB or VTA (Figure 2G). SEAR in the ACx was maximally reduced when the stimulation of the NB or VTA coincided with the acoustic stimulus (NB by 57.3% ± 8.6%, p = 0.002; VTA by 49.2% ± 3.9%, p < 0.001) (Figures 2H and 2I). SEAR was unaltered when the stimulation of the NB or VTA and the acoustic stimulus were separated by more than 5 s (p > 0.05) (Figures 2H and 2I). In agreement with the notion that neuromodulatory inputs mediate the US information during associative cortical plasticity,^19^ pairing an aversive stimulus (mild tail shock) with an acoustic stimulus reduced SEAR in the ACx by 44.9% ± 4.3% (p = 0.0003) relative to baseline (Figures 2J and 2K). In contrast, SEAR was unaffected when the tail shock preceded an acoustic stimulus by 20 s (Figure 2K).

### NT5E overexpression in the auditory thalamus prevents SEAR reduction below the threshold permissive for TC synaptic plasticity and associative cortical plasticity

**Figure 3.**
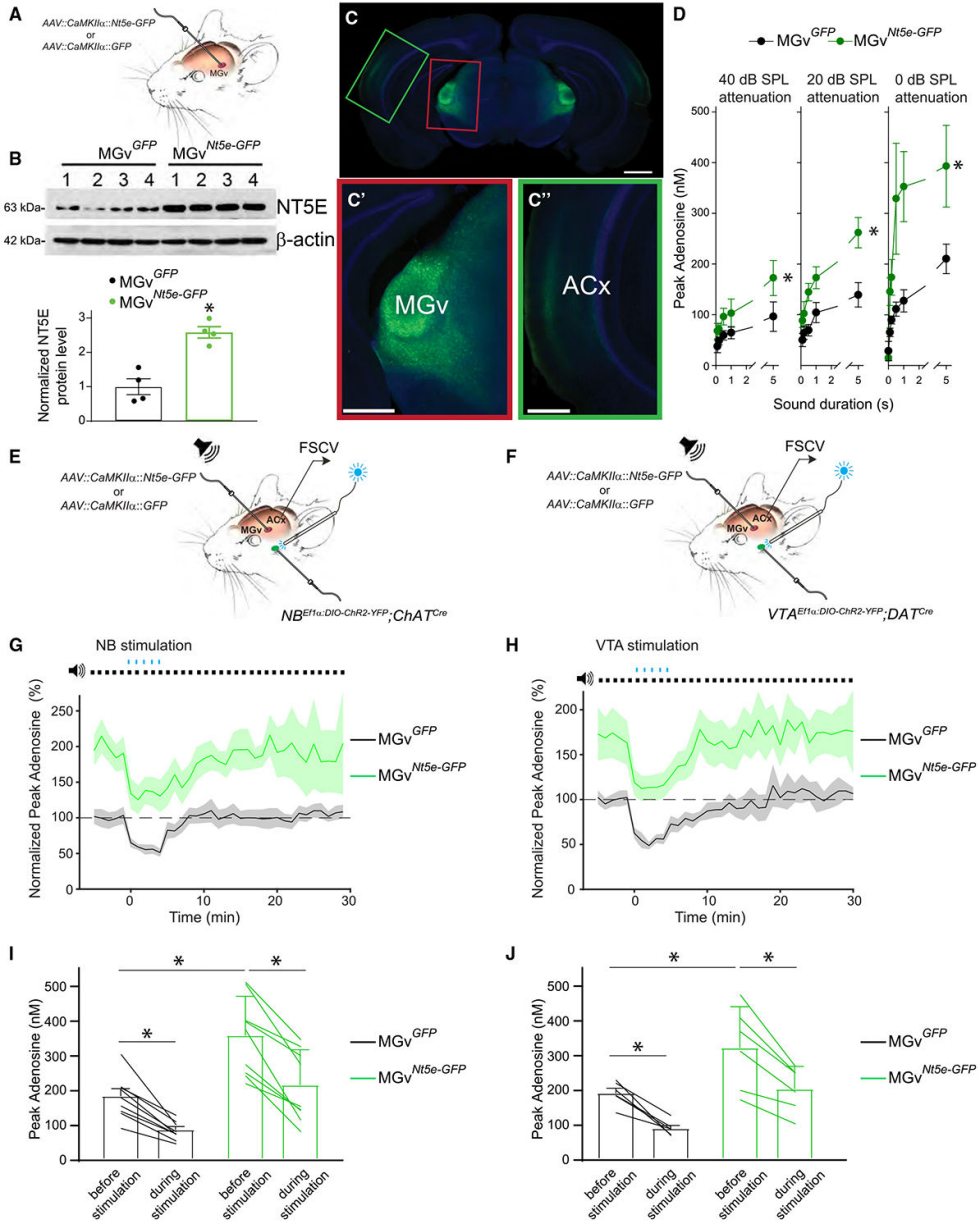
NT5E overexpression in the MGv prevents the low-adenosine condition in the ACx (A) Overexpression of NT5E in the MGv by *in vivo* viral injection of *AAV-CaMKIIa-Nt5e-GFP* in WT mice (*MGv*^*Nt5e-GFP*^ mice). *AAV-CaMKIIa*-GFP-injected WT mice were controls (*MGv*^*GFP*^ mice). (B) Western blot of MGv extracts (top) and mean NT5E levels (bottom) in the MGv (unpaired 2-tailed t test, *t*_*6*_ = 5.56, *p = 0.0014) of *MGv*^*Nt5e-GFP*^ mice (n = 4) or *MGv*^*GFP*^ mice (n = 4). (C–C") Low-magnification (C) and high-magnification (C′, C″) images of a TC slice illustrating GFP expression in the MGv (C′) of *MGv*^*Nt5e-GFP*^ mice. Weak expression of GFP is also detected in the thalamorecipient layer of the ACx, indicative of labeled TC projections (C″). Scale bars, 1 mm (C) and 0.5 mm (C′ and C″). (D) Peak adenosine concentration as a function of sound duration in *MGv*^*Nt5e-GFP*^ mice and *MGv*^*GFP*^ mice exposed to broadband noise with 40-dB (left, 4 *MGv*^*GFP*^ mice, 5 *MGv*^*Nt5e-GFP*^ mice), 20-dB (center, 4 *MGv*^*GFP*^ mice, 5 *MGv*^*Nt5e-GFP*^ mice), and 0-dB (right, 7 *MGv*^*GFP*^ mice, 9 *MGv*^*Nt5e-GFP*^ mice) SPL (3-way ANOVA, genotype × attn, *F*_*1,2*_ = 4.6, *p = 0.01, genotype *F*_*1*_ = 25.4, *p < 0.001). (E and F) Diagrams of measuring extracellular adenosine in the ACx by pairing pure tones (speaker) with optogenetic activation of the NB (*NB*^*ChR2*^ mice, E) and VTA (*VTA*^*ChR2*^ mice, F) projections in the ACx. The NB or VTA was activated using 470-nm light in *MGv*^*Nt5e-GFP*^ or *MGv*^*GFP*^ mice. (G and H) Normalized mean peak of SEAR in the ACx was measured in response to broadband noise (5 s) before, during, and after pairing it with NB (G) or VTA (H) optical stimulation (5 blue dots) in control *MGv*^*GFP*^ mice (NB, n = 9; VTA, n = 6) and *MGv*^*Nt5e-GFP*^ mice (NB, n = 9; VTA, n = 6). Data are normalized to the baseline recorded in *MGv*^*GFP*^ mice (dotted line). (I and J) Mean peak adenosine measured in the ACx in response to 5-s broadband noise before and during optogenetic stimulation of the NB (I) or VTA (J) in *MGv*^*Nt5e-GFP*^ or *MGv*^*GFP*^ mice. NB: *MGv*^*GFP*^ (n = 9) and *MGv*^*Nt5e-GFP*^ (n = 9): 1-way ANOVA, *F*_*3*_ = 16.7, *p < 0.001. Holm-Sidak post hoc method: *MGv*^*GFP*^ before vs. during stimulation, *p = 0.035; *MGv*^*Nt5e-GFP*^ before vs. during stimulation, *p = 0.003; *MGv*^*GFP*^ before stimulation vs. *MGv*^*Nt5e-GFP*^ before stimulation, *p < 0.001; *MGv*^*GFP*^ before stimulation vs. *MGv*^*Nt5e-GFP*^ during stimulation: p = 0.425. VTA: *MGv*^*GFP*^ (n = 6) and *MGv*^*Nt5e-GFP*^ (n = 6): 1-way ANOVA, *F*_*3*_ = 10.9, *p < 0.001. Holm-Sidak post hoc method: *MGv*^*GFP*^ before vs. during stimulation, *p = 0.042; *MGv*^*Nt5e-GFP*^ before vs. during stimulation, *p = 0.034; *MGv*^*GFP*^ before stimulation vs. *MGv*^*Nt5e-GFP*^ before stimulation, *p < 0.022; *MGv*^*GFP*^ before stimulation vs. *MGv*^*Nt5e-GFP*^ during stimulation: p = 0.77. Averaged data are presented as the mean ± SEM.

Pairing an acoustic stimulus with neuromodulatory activation transiently reduces SEAR and induces ACx plasticity. Therefore, we asked whether the dip in TC SEAR is necessary for cortical plasticity. To answer this question, we prevented the adenosine level from decreasing below a presumed plasticity-permissive threshold by overexpressing NT5E in thalamic excitatory neurons of the MGv. To that end, we injected *AAV-CaMKIIα-Nt5e-GFP* (or control *AAV-CaMKIIα-GFP*) bilaterally into the MGv (Figure 3A). The NT5E level increased ~2.5-fold (p = 0.001) in the MGv of mice injected with *AAV-CaMKIIα-Nt5e-GFP (MGv*^*Nt5e-GFP*^ mice) compared with that in mice injected with *AAV-CaMKIIα-GFP* (*MGv*^*GFP*^ mice) (Figure 3B), and *Nt5e-GFP* was expressed only in the MGv (Figure 3C). Broadband noise of various durations and intensities induced significantly higher SEAR (p < 0.001) in *MGv*^*Nt5e-GFP*^ mice than in *MGv*^*GFP*^ mice (Figure 3D). On average, SEAR induced by 5-s sounds increased by 83.0% ± 2.1% in *MGv*^*Nt5e-GFP*^ mice (p < 0.001) relative to that in control *MGv*^*GFP*^ mice. As in WT mice, NB- or VTA-sound pairings reduced SEAR in *MGv*^*Nt5e-GFP*^ mice (Figures 3E-3J). We paired broadband noise with optogenetic stimulation (470 nm) of NB or VTA projections to the ACx in *NB*^*ChR2*^ mice or in *VTA*^*ChR2*^ mice, respectively, with overexpression of NT5E or GFP in the MGv (Figures 3E and 3F). Baseline SEAR levels were higher in *NB^ChR2^;MGv^Nt5e-GFP^* mice than in *NB^ChR2^;MGv^GFP^* mice, and they were higher in *VTA^ChR^;MGv^Nt5e-GFP^* mice than in *VTA^ChR2^;MGv^GFP^* mice. Cholinergic or dopaminergic stimulation in these NT5e-overexpressing mice did not reduce SEAR to the extent seen in the respective control mice (Figures 3G-3J).

**Figure 4.**
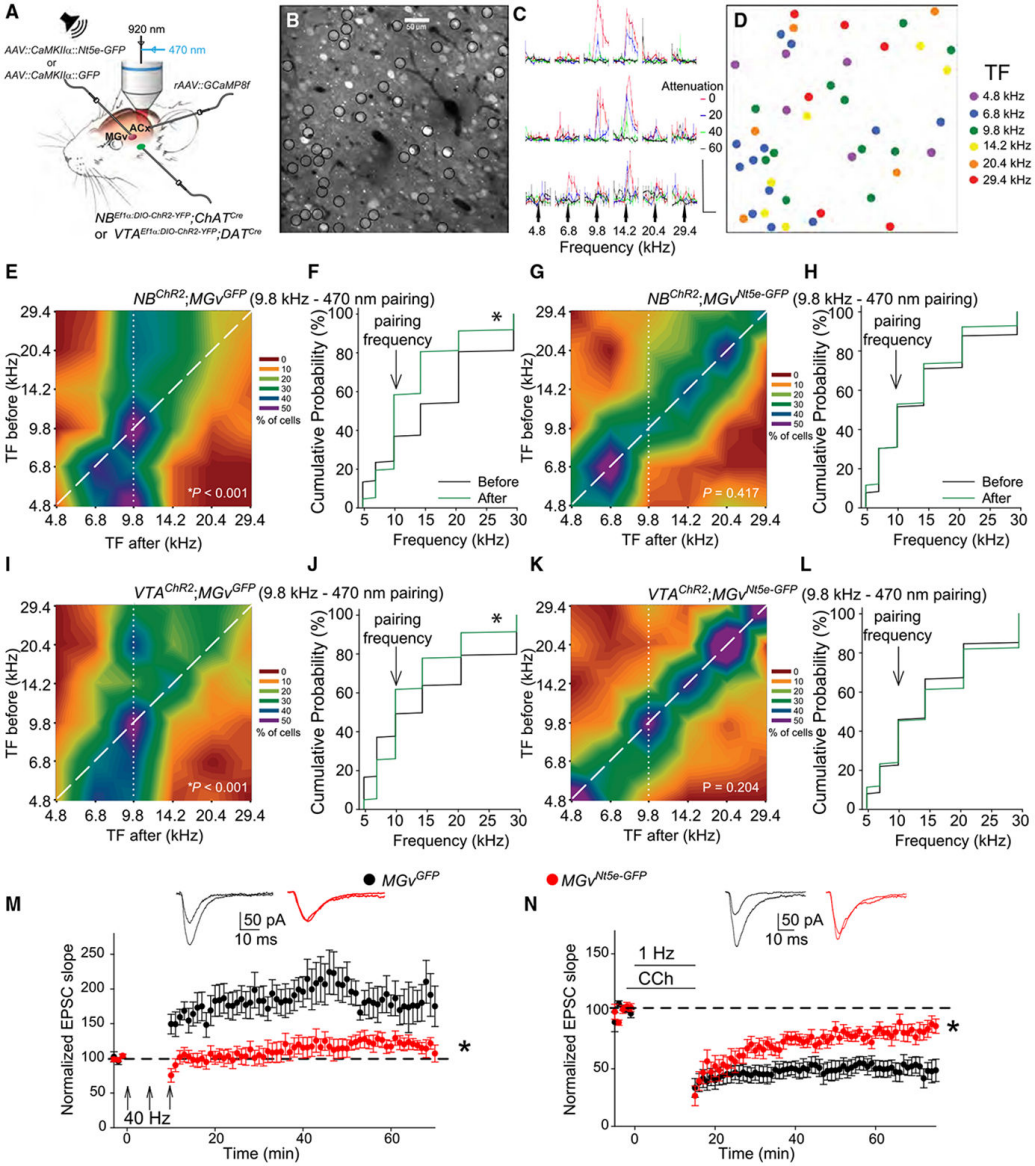
NT5E overexpression in the MGv prevents ACx plasticity in adult mice (A) Diagram of measuring tuning frequency (TF) plasticity via 2-p calcium imaging of GCaMP8f fluorescence (at 920 nm) in the ACx. Pure tones were paired with optogenetic activation of the NB (*ChAT*^*Cre*^ mice) or VTA (*DAT*^*Cre*^ mice) projections in the ACx by using 470-nm light in *MGv*^*Nt5e-GFP*^ or *MGv*^*−GFP*^ mice. (B) Image of jGCaMP8f-expressing excitatory neurons in the ACx. Circles identify sound-responsive neurons. Scale bar, 50 μm. (C) Each row shows ΔF/F trace averages from a different neuron during sound stimulation, ranging from a narrowly tuned cell (top row) to a more broadly tuned cell (bottom row). Traces are sorted to frequency and level of attn (in dB SPL) and averaged over 75 trails. Arrows show the timing of sound stimulation. Fluorescence was sampled at 10 frames/s. Scale bar, 50% ΔF/F, 500 ms. (D) Map of sound-responsive neurons (from B, circled cells), with their respective TFs indicated. (E–L) Heatmaps (ΔTF maps) depicting shifts in neuron numbers (normalized to percent sampled) (E, G, I, and K) and cumulative histograms of TFs of recorded neurons in the ACx (F, H, J, and L) before and after pairing a pure tone (9.8-kHz) with optogenetic stimulation of NB projections in *ChAT*^*Cre*^ mice injected with *AAV-Ef1a-DIO-ChR2-YFP* (*NB*^*ChR2*^ mice) (E–H) or before and after pairing a 9.8-kHz pure tone with optogenetic stimulation of VTA projections in *DAT*^*Cre*^ mice injected with *AAV-Ef1a-DIO-ChR2-YFP* (*VTA*^*ChR2*^ mice) (I–L) in *MGv*^*GFP*^ (E, F, I, and J) or *MGv*^*Nt5e-GFP*^ mice (G, H, K, and L). Diagonal lines represent no change in TFs after pairing, and vertical lines represent 9.8 kHz (pairing frequency). (E and F) 149 neurons, 3 mice, Wilcoxon signed-rank test *Z* = −6.00, *p < 0.001 (E) and Kolmogorov-Smirnov (K-S) test *D* = 0.27, *p < 0.001 (F). (G and H) 155 neurons, 3 mice, Wilcoxon signed-rank test *Z* = −0.82, p = 0.417 (G) and K-S test *D* = 0.04, p = 0.997 (H). (I and J) 199 cells, 3 mice, Wilcoxon-signed rank test *Z* = −5.6, *p < 0.001 (I) and K-S test *D* = 0.14, *p = 0.035 (J). (K and L) 150 cells, 3 mice, Wilcoxon signed-rank test *Z* = 1.27, p = 0.204 (K) and K-S test *D* = 0.05, p = 0.980 (L). (M and N) TC LTP and TC LTD are impaired in *MGv*^*Nt5e-GFP*^ mice. Mean, normalized slope of excitatory postsynaptic current (EPSC) as a function of time before and after induction of TC LTP (M) or TC LTD (N) in *MGv*^*Nt5e-GFP*^ mice (LTP, n = 15; LTD, n = 11) and *MGv*^*GFP*^ mice (LTP, n = 12; LTD, n = 10). Insets show representative EPSCs recorded before and after induction of TC LTP or TC LTD. CCh, carbachol (5 μM). Dashed lines indicate the baseline before induction of TC LTP or LTD. LTP in *MGv*^*GFP*^ vs. *MGv*^*Nt5e-GFP*^ mice: Mann-Whitney rank-sum test, *U* = 25, *p = 0.002. LTD in *MGv*^*GFP*^ vs. *MGv*^*Nt5e-GFP*^ mice: unpaired t test, *t*_*19*_ = −3.847, *p = 0.001. Data are presented as the mean ± SEM.

Next, we sought to verify the link between the adenosine level in the MGv and cortical plasticity. Two-photon jGCaMP8f calcium imaging of excitatory neurons in the thalamorecipient layer (L) 3/4 of the ACx (Figures 4A-4D and S6A) revealed that pairing a 9.8-kHz pure tone with electric or optogenetic stimulation of cholinergic neurons in the NB or their projections in the ACx induced cortical plasticity of receptive fields (in the form of stimulus-specific tuning frequency [TF] shifts) in individual neurons in control (*MGv*^*GFP*^)mice(Figures 4E, 4F, S6B, and S6C). In these experiments, we determined each neuron’s TF from calcium transients evoked by various tone frequencies and intensities (Figures 4C and 4D).^55^ We measured changes in TFs by imaging the same neurons before and after pairing a 9.8-kHz tone with NB stimulation. When we paired a 9.8-kHz tone with optogenetic stimulation of the NB in WT mice and in *NB*^*ChR2*^ mice, the TF of sound-responsive ACx neurons shifted toward 9.8 kHz (Figures 4E and 4F). This shift was observed in each mouse tested within this *NB^ChR2^;MGv^GFP^* group (Figure S7A). Although TFs shifted toward 9.8 kHz in *NB^ChR2^;MGv^GFP^* mice after 9.8 kHz-470 nm pairing, this plasticity was eliminated in all *NB^ChR2^;MGv^Nt5e-GFP^* mice (Figures 4G, 4H, and S7B). Similarly, when we paired a 9.8-kHz tone with electrical stimulation of the NB in WT (*MGv*^*GFP*^) mice, the TF of sound-responsive ACx neurons shifted toward 9.8 kHz in every mouse tested (Figure S6D), and this TF plasticity was eliminated in all *MGv*^*Nt5e-GFP*^ mice (Figures S6E-S6G). NT5E overexpression in the MGv also blocked cortical TF plasticity when we paired a pure tone with optogenetic stimulation of VTA projections in the ACx. In *VTA^ChR2^;MGv^GFP^* mice, TFs shifted toward 9.8 kHz when we paired a 9.8-kHz tone with optogenetic stimulation of VTA projections in the ACx (Figures 4I and 4J), and this shift was observed in every mouse tested (Figure S7C). However, this shift was eliminated in all *VTA^ChR2^;MGv^Nt5e-GFP^* mice (Figures 4K, 4L, and S7D).

Consistent with the notion that long-term synaptic plasticity at TC projections is a cellular correlate of ACx plasticity,^58,66,67^ NT5E overexpression in the MGv significantly impaired TC LTP and LTD (Figures 4M and 4N). TC LTP induced by 40-Hz tetanization of the thalamic radiation, which excites both TC projections and cholinergic projections,^67^ produced TC LTP in *MGv*^*GFP*^ mice, which was substantially reduced in *MGv*^*Nt5e-GFP*^ mice (~90% decrease comparatively, p = 0.002). Similarly, pairing 1-Hz stimulation of the TC projections with application of the cholinergic agonist carbachol produces TC LTD in *MGv*^*GFP*^ mice^66^; this LTD was ~65% attenuated in *MGv*^*Nt5e-GFP*^ mice (p = 0.001).

### Thalamic NT5E overexpression prevents auditory perceptual plasticity and learning and memory

**Figure 5.**
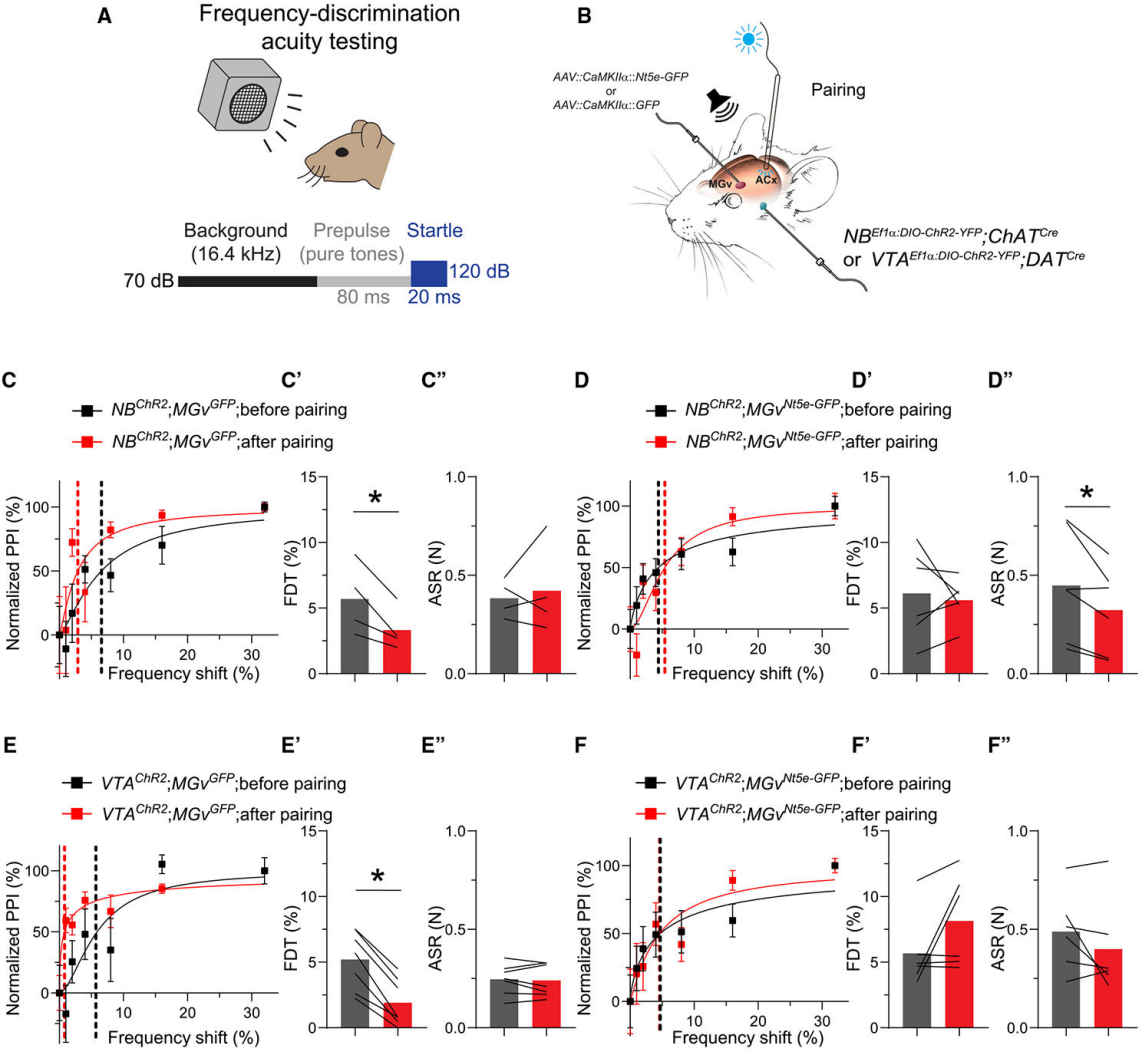
Long-term synaptic plasticity at TC projections and auditory perceptual plasticity are occluded by elevated adenosine in the MGv of adult mice (A) Schematic of testing frequency discrimination acuity. A background tone (16.4 kHz, 70-dB SPL) is present throughout the experiment. Variable frequency prepulse tones (80-ms, 70-dB SPL) are presented prior to the startle stimulus (white noise, 20-ms, 120-dB SPL). (B) Schematic showing the NB- or VTA-sound pairing procedures. Optogenetic stimulation of the NB or VTA projections in the ACx was paired with a pure tone (16.4 or 8.2 kHz, 70-dB SPL) in *MGv*^*GFP*^ or *MGv*^*Nt5e-GFP*^ mice. (C–D″) Improved auditory perception induced in *MGv*^*GFP*^ mice by optogenetic NB-16.4-kHz pairing (C–C″) is prevented in *MGv*^*Nt5e-GFP*^ mice (D–D″). NB projections were activated optogenetically in the ACx after (*AAV-Ef1a-DIO-hChR2(E123T/T159C)-EYFP* was injected into the basal forebrain of *ChAT*^*Cre*^ mice. (C–C") Representative recordings from a mouse with normalized PPI magnitude, as a function of frequency difference between background and prepulse tones, before and after optogenetic NB-16.4-kHz pairing. Points and error bars are mean ± SEM of 10 repeated measurements in the same animal. Solid lines are logistic regression fits to these points; dotted lines are FDTs. (C′) The FDT decreases after optogenetic NB-16.4-kHz pairing; 2-tailed paired t test, *t*_*3*_ = 3.47, *p = 0.04 (n = 4 mice). (C″) The ASR is unchanged after optogenetic NB-16.4-kHz pairing; 2-tailed paired t test, *t*_*3*_ = 0.46, p = 0.678 (n = 4 mice). (D–D″) Representative recordings of normalized PPI magnitude, as a function of frequency difference between background and prepulse tones, before and after optogenetic NB-16.4-kHz pairing (D), FDT is unchanged, 2-tailed paired t test, *t*_*5*_ = 0.457, p = 0.667 (n = 6 mice) (D′), and ASR decreases, 2-tailed paired t test, *t*_*5*_ = 2.81, *p = 0.038 (n = 6 mice) (D″), in *NB^ChR2^;MGv^Nt5e-GFP^* mice. (E–F″) Improved auditory perception induced in *MGv*^*GFP*^ mice by optogenetic VTA-16.4-kHz pairing (E–E″) is prevented in *MGv*^*NtSe-GFP*^ mice (F–F″). VTA projections were activated optogenetically in the ACx after (*AAV-Ef1a-DIO-hChR2(E123T/T159C)-EYFP* was injected into the basal forebrain of *ChAT*^*Cre*^ mice. (E–E″) Representative recordings of normalized PPI magnitude, as a function of frequency difference between background and prepulse tones, before and after optogenetic VTA-16.4-kHz pairing (E), (E’) FDT decreases, 2-tailed paired t test, *t*_*6*_ = 8.391, *p = 0.0002 (n = 7 mice). (E″) The ASR is unchanged, 2-tailed paired t test, *t*_*6*_ = 0.439, p = 0.677 (n = 7 mice) in *VTA^ChR2^;MGv^GFP^* mice. (F–F″) Representative recordings of normalized PPI magnitude as a function of frequency difference between background and prepulse tones, before and after optogenetic VTA-16.4-kHz pairing (F). (F’) The FDT is unchanged, 2-tailed paired t test, *t*_*5*_ = 1.82, p = 0.128 (n = 6 mice). (F″) The ASR is unchanged, 2-tailed paired t test, *t*_*5*_ = 1.38, p = 0.226 (n = 6 mice) (F″), in *VTA^ChR2^;MGv*Nt5e-GFP** mice. Data are presented as the mean ± SEM.

We then tested whether NT5E overexpression in the MGv affects auditory perceptual performance. NB- or VTA-sound pairings improved frequency discrimination acuity in control mice. We called this experience-dependent shift in frequency discrimination acuity “auditory perceptual plasticity.” We measured frequency discrimination acuity using a test based on pre-pulse inhibition (PPI) of the acoustic startle response (ASR) (Figures 5A and S8A). PPI is proportional to the magnitude of the frequency difference between the pre-pulse tone and the background frequency (16.4 kHz) prior to a brief, loud noise.^77^ When we paired a 16.4-kHz tone with electrical stimulation of the NB or VTA (Figure S8B), mice exhibited significantly greater PPI at pre-pulse frequencies closer to background than before the respective pairings (Figures S8C and S8D). We used a decreased frequency discrimination threshold (FDT),^11,77,78^ the pre-pulse frequency that yielded 50% of the maximal PPI, as an indicator of auditory perceptual plasticity. FDT after NB- or VTA-16.4-kHz pairing was, on average, ~60% lower than before pairing (Figure S8C′ and S8D′), and ASR was unchanged (Figures S8C″ and S8D″).

NB- or VTA-8.2-kHz pairing did not affect FDT (Figures S8E-S8E″ and S8F-S8F″) when we tested frequency discrimination relative to 16.4 kHz, by stimulation of the NB or VTA alone (Figures S8G-S8G″ and S8H-S8H″), or by PPI tests on 2 consecutive days (Figures S8I-S8I″).

NT5E overexpression in the MGv occluded the auditory perceptual plasticity induced by electrical NB- or VTA-16.4-kHz pairing (Figures S9A and S9B). FDT after NB-16.4-kHz pairing was ~50% lower (p = 0.025) than before the pairing, with no change in the ASR in control *MGv*^*GFP*^ mice (Figures S9C-S9C″). However, FDT remained unchanged after NB-16.4-kHz pairing in *MGv*^*Nt5e-GFP*^ mice (Figures S9D-S9D″). Similarly, after VTA-16.4-kHz pairing, FDT was reduced ~60% (p < 0.0001), and ASR was unchanged in *MGv*^*GFP*^ mice (Figures S9E-S9E″). However, FDT was unchanged after VTA-16.4-kHz pairing in *MGv*^*Nt5e-GFP*^ mice (Figures S9F-S9F″).

NT5E overexpression in the MGv also occluded the auditory perceptual plasticity induced by optogenetic NB- or VTA-16.4-kHz pairing (Figure 5B). Although frequency discrimination acuity improved after pairing a 16.4-kHz tone with illumination of the ACx at 470 nm in *NB^ChR2^;MGv^GFP^* mice and *VTA^ChR2^;MGv^GFP^* mice, this improvement was eliminated in *NB^ChR2^;MGv^Nt5e-GFP^* mice and *VTA^ChR2^;MGv^Nt5e-GFP^* mice (Figures 5C-5F″). After 16.4-kHz-470-nm pairing, FDT was reduced ~40% (p = 0.04) compared with that before pairing, and the ASR was unchanged in *NB^ChR2^;MGv^GFP^* mice (Figures 5C-5C″). However, this protocol failed to reduce FDT in *NB^ChR2^;MGv^Nt5e-GFP^* mice (Figures 5D-5D″). In a similar fashion, 16.4-kHz-470-nm pairing lowered FDT ~70% (p = 0.0002) in *VTA^ChR2^;MGv^GFP^* mice without changing the ASR (Figures 5E-5E″). The same protocol failed to reduce FDT in *VTA^ChR2^;MGv^Nt5e-GFP^* mice (Figures 5F-5F″).

**Figure 6.**
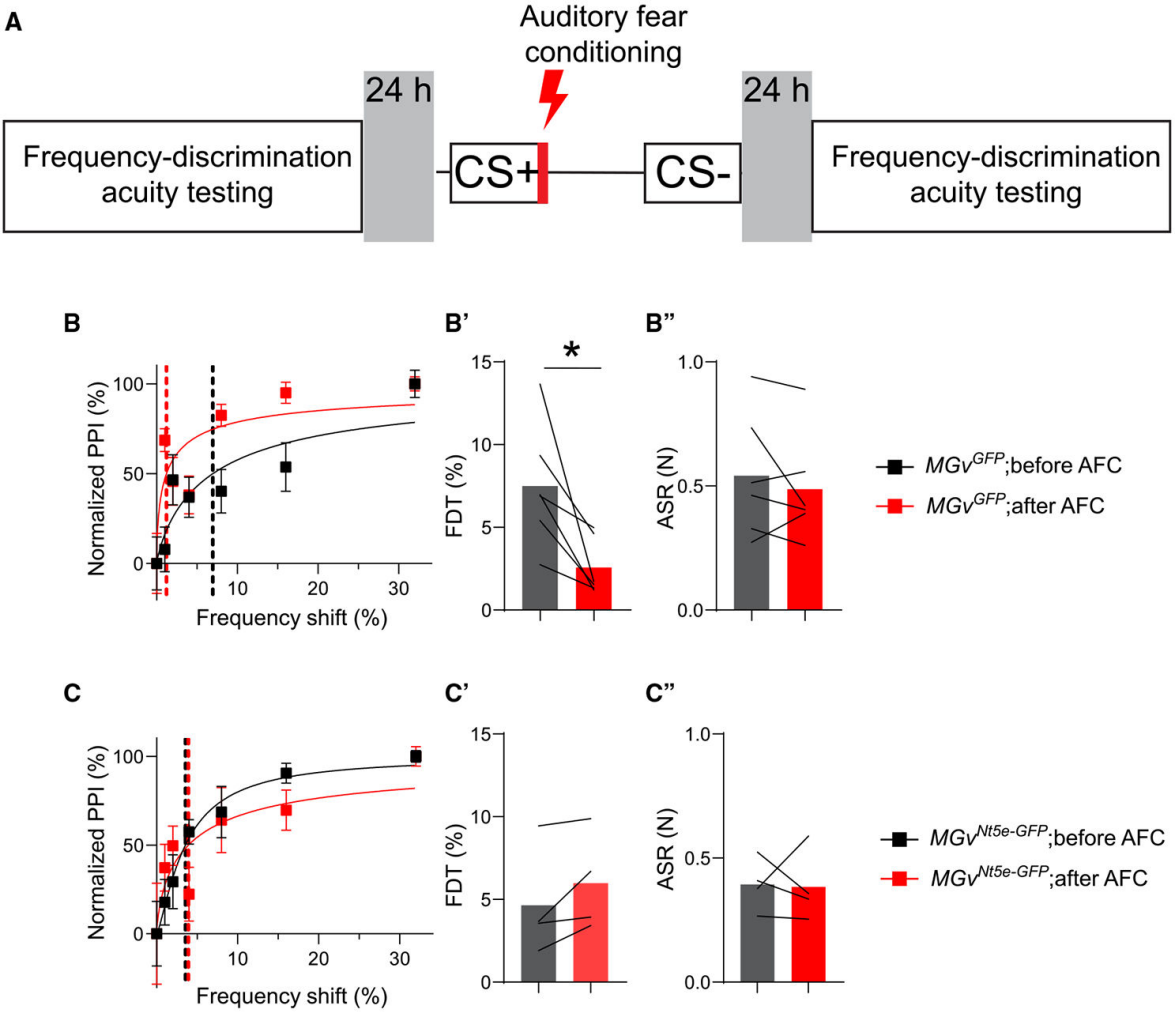
Auditory perceptual learning and memory induced by AFC is prevented by the elevated thalamic adenosine level (A) Schematic of testing auditory perceptual learning and memory. Frequency discrimination acuity was tested 24 h before and 24 h after the auditory fear conditioning (AFC) protocol. AFC sessions consisted of two 80-db SPL pure tones presented 8 times each for 20.5 s, alternating between 16.4 kHz (CS+) co-terminating with a mild shock (0.5 s, 0.5 mV) and 8.2 kHz (CS−, coarse fear conditioning) or 13.94 kHz (CS−, fine fear conditioning). (B–B″) AFC induced perceptual learning and memory in control (*MGv*^*GFP*^) mice (B). Shown are representative recordings from a mouse with normalized PPI magnitude, as a function of frequency difference between background and prepulse tones, before and after AFC. Points and error bars are mean ± SEM of 10 repeated measurements in the same animal. Solid lines are logistic regression fits to these points; dotted lines are FDTs. The FDT decreased after AFC; 2-tailed paired t test, *t*_*5*_ = 3.174, *p = 0.025 (n = 6 mice) (B′). The ASR was unchanged after AFC in *MGv*^*GFP*^ mice; 2-tailed paired t test, *t*_*5*_ = 0.919, p = 0.4 (n = 6 mice) (B″). (C–C″) Perceptual learning and memory induced by AFC did not occur in mice with elevated adenosine levels in the auditory thalamus (*MGv*^*Nt5e-GFP*^ mice). (C) Example of recordings from an *MGv*^*Nt5e-GFP*^ mouse; normalized PPI magnitude is shown as a function of frequency difference between background and prepulse tones, before and after AFC. Points and error bars are mean ± SEM of 10 repeated measurements in the same animal. Solid lines are 3-parameter logistic regression curves fit to these points; dotted lines are FDTs. (C′ and C″) No change was detected in FDT (2-tailed paired t test, *t*_*3*_ = 2.154, p = 0.120, n = 4 mice, C′) or in the ASR (2-tailed paired t test, *t*_*3*_ = 0.129, p = 0.906, n = 4 mice, C″) before or after AFC. Data are presented as the mean ± SEM.

We then assessed whether learning the significance of certain sounds would produce a long-term change in auditory perception. We tested frequency discrimination acuity before and 24 h after subjecting mice to auditory fear conditioning (AFC), during which they learned to differentiate between 16.4 kHz (CS+) and 8.2 kHz (CS−) and between 16.4 kHz (CS+) and 13.94 kHz (CS−) (Figure 6A). Consistent with previous results,^78^ AFC improved frequency discrimination acuity in *MGv*^*GFP*^ mice (Figures 6B and 6B′) without affecting the ASR (Figure 6B″), indicating that control mice have robust experience-dependent auditory perceptual memory. However, *MGv*^*Nt5e-GFP*^ mice with elevated adenosine levels in the MGv showed no effect of AFC on FDT or ASR (Figures 6C-6C″).

## DISCUSSION

Here we describe the SEAR phenomenon in mice. During SEAR, acoustic stimuli cause a rise in extracellular adenosine, a negative regulator of neurotransmitter release,^56-58^ from glutamatergic TC projections to the ACx. SEAR is an activity-dependent phenomenon that occurs only in awake animals, is triggered by acoustic stimuli, and relies on the duration and intensity of that stimulus.

Our data suggest that SEAR dynamics mediate a fundamental mechanism of cortical plasticity and perceptual learning in the adult brain, in which representations of sensory information are stored during episodes of heightened behavioral importance mediated by a transient low-adenosine condition. Experience-dependent improvement in auditory perception serves for better segregation of predatorial or threatening sounds from safe sounds in a dynamic environment and represents a form of auditory perceptual learning and memory.^79-81^ Unencumbered SEAR elevates the level of adenosine, which, being a negative regulator of synaptic plasticity at glutamatergic synapses,^47,58,82^ targets presynaptic A_1_Rs on TC projections, prevents cortical plasticity induced by sound stimulation alone, and contributes to the closure of the early critical period of cortical plasticity in the ACx.^47,55^ Here we show that this adenosine-dependent mechanism permits auditory learning and memory in adults by neuromodulatory circuits dynamically reducing SEAR.

A salient stimulus or neuromodulatory projections that telegraph elevated attention, vigilance, and alertness, both of which assign behavioral importance to incoming sensory information, transiently impair SEAR, thereby producing a low-adenosine condition in the ACx during periods of attentive wakefulness. These results support the notion of SEAR as a mechanism of convergence between the CS and US during associative cortical plasticity and learning.

The CS-US convergence site has been proposed previously at the level of the excitatory/inhibitory balance, as the phasic release of neuromodulators operates through disinhibition of local inhibitory microcircuits to enhance the excitability of CS-activated cortical pyramidal neurons.^11,22,29,36,49,83,84^ Here, we show that the CS-US convergence occurs at the presynaptic locus of excitatory TC projections and that this convergence reduces SEAR, thereby producing a low-adenosine condition akin to that seen during the early critical period in pups.^47,55^

The low-adenosine condition occurs in a highly associative manner and can be produced by pairing a sound stimulus with either tail shock or neuromodulatory circuit activation. SEAR is maximally reduced when the 2 stimuli temporarily coincide, and SEAR is unaffected when more than 10 s elapses between the 2 stimuli. This points to SEAR as a gatekeeper of ACx plasticity, and the convergence of the US (encoded by the neuromodulatory circuits) with the CS (transmitted by TC projections to the ACx) removes this gate, enabling sensory stimuli to induce cortical plasticity in the adult ACx.^47,55^ In agreement with this notion, elevating the adenosine level in the auditory thalamus occluded cortical plasticity in the ACx and prevented long-term TC synaptic plasticity, which is thought to be the cellular correlate of cortical plasticity in the ACx,^54,58,66,67,85^ auditory perceptual plasticity, and auditory learning.

Together, these results suggest that the transient low-adenosine condition during the presentation of auditory stimuli is a necessary component of cortical plasticity in the adult ACx and auditory learning and memory. The exact mechanisms of adenosine production, release, and decrease during neuromodulatory circuit activation will need to be elucidated in future studies. Although astroglia have been implicated as a source of adenosine,^86,87^ we determined that the major source of SEAR is TC glutamatergic projections. Thus, adenosine is most likely produced when thalamic projections co-release ATP and glutamate, as described for other neurons.^88-90^

ATP is cleaved to ADP and then to AMP to form adenosine via the specific NT5E-mediated mechanism.^91-93^ The relatively quick rise time of SEAR is consistent with ATP metabolism being extremely rapid. ATP, ADP, and AMP are converted to adenosine, and the half-time of ATP conversion to adenosine is ~200 ms.^94^ Activation of cholinergic or dopaminergic circuits in the ACx removes sound-evoked extracellular adenosine within seconds. Our present and previous data^66,67^ indicate that M1 muscarinic or D2/D3 dopaminergic receptors, respectively, mediate this process. However, the cellular location of these receptors and the machinery of neuromodulator-dependent adenosine removal has yet to be established.

In future studies, elucidating the subcellular localization of the neuromodulator receptors and the mechanism of neuromodulator-triggered SEAR decrease in the ACx will be essential. Furthermore, determining not only whether other stimuli (sensory or otherwise) release adenosine at their respective targets but also whether plasticity in those targets requires dynamic reduction of stimulus-evoked adenosine release by neuromodulatory circuits will be imperative. This approach may be generalized toward understanding a fundamental, adenosine-based paradigm in associative plastic changes that occur in the adult brain during stimuli of behavioral importance.

**Figure 7.**
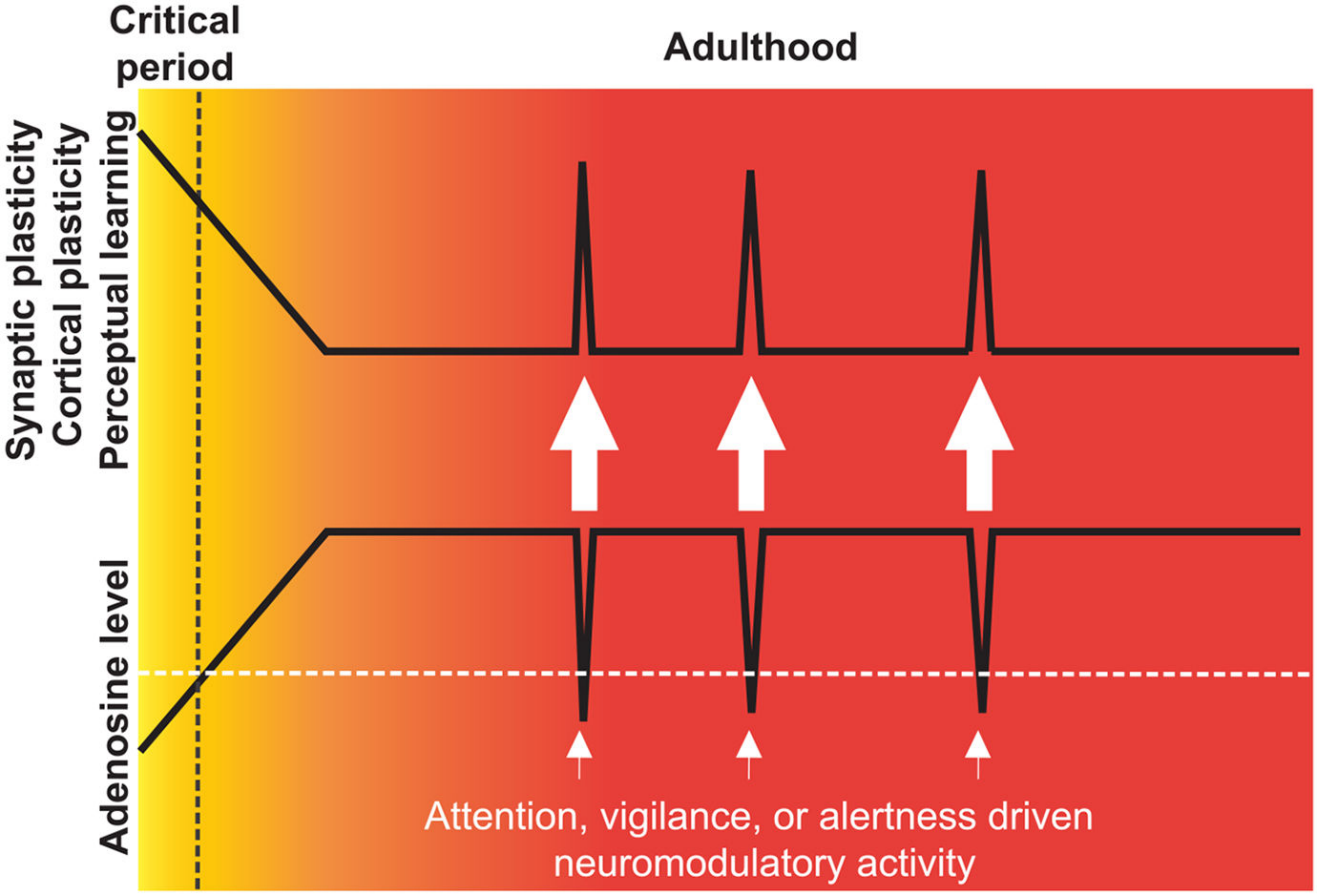
Model of adenosine’s role in cortical plasticity and auditory perceptual learning throughout development Low adenosine levels during the early critical period promote cortical plasticity, perceptual plasticity, and auditory learning by sensory experience alone. High adenosine levels (in part supplied by SEAR) in adults restrict cortical plasticity and perceptual learning, except when adults are in the state of attentive wakefulness. Periods of attentive wakefulness activate neuromodulatory circuits (small arrows), which, in turn, reduce adenosine to the low (permissive) level (horizontal dashed line). These transient low-adenosine conditions allow synaptic plasticity, cortical plasticity, and auditory perceptual learning to occur (large arrows) in response to sensory experience.

In conclusion, here we describe a fundamental mechanism of auditory associative cortical plasticity and perceptual learning in adults: transient low-adenosine episodes brought about by activation of neuromodulatory circuits enabled the storage of behaviorally important sensory information. We report two major findings. First, acoustic stimuli evoke adenosine release from glutamatergic TC projections to the ACx in awake mice. Second, the activation of neuromodulatory projections transiently impairs SEAR in a tightly coordinated manner, thereby producing a seconds-long early critical period-like condition. This low-adenosine condition in the auditory thalamus is required for sensory stimuli to induce associative stimulus-specific cortical plasticity and enable auditory learning (Figure 7). These results reveal SEAR as a dynamic gatekeeper of brain plasticity. A seconds-long low-adenosine window may act as an eligibility trace, a sustained memory of recent activity that renders synaptic connections malleable to modification over several seconds,^95^ or as a synaptic memory trace for sensory information of heightened significance. The latter would allow specific sensory stimuli to induce long-term synaptic plasticity at TC synapses and readjust receptive fields in the sensory cortices to represent the newly acquired importance of these sensory stimuli.^19,96^ These results also suggest that, during stimuli (sensory or otherwise) of behavioral importance, the adult brain switches to the juvenile-like state by briefly decreasing the adenosine concentration to levels permissive to induce experience-dependent plasticity and learning.

### Limitations of the study

The FSCV method used was selective for adenosine; it did not detect ATP, ADP, AMP, neuromodulators, nucleotides, or nucleosides, except for adenine, which has the same electroactive moiety as adenosine. This may call into question the specificity of this method. However, we argue that the FSCV signal we see *in vivo* in response to sounds is adenosine, not adenine. To our knowledge, adenine is not present extracellularly in the brain, nor do neurons release adenine in an activity-dependent manner. Moreover, the GRAB_Ado_ fluorescent indicator is sensitive to adenosine but not adenine, and there is a strong correlation (*r*^*2*^ = 0.984; p = 0.008) between the FSCV and GRAB_Ado_ responses to adenosine. Furthermore, the FSCV signatures of adenosine and adenine differ, and sound-evoked FSCV responses *in vivo* resemble those of adenosine, not adenine. Although this strongly argues that acoustic stimulation produces SEAR in the ACx, other unidentified compounds may contaminate the FSCV responses *in vivo*. Therefore, using two complimentary methods, FSCV and GRAB_Ado_, ensured the accurate detection of extracellular adenosine *in vivo*.

## STAR★METHODS

### RESOURCE AVAILABILITY

#### Lead contact

Further information and requests for resources and reagents should be directed to and will be fulfilled by the lead contact, Stanislav S. Zakharenko (stanislav.zakharenko@stjude.org).

#### Materials availability

*Nt5e*^*fl/+*^ mice or viruses generated in this study are available from the lead contact upon request.

#### Data and code availability

- All data are available upon request from the lead contact.
- This paper does not report any original code.
- Any additional information required to re-analyze the data reported in this paper is available from the lead contact upon request.

### EXPERIMENTAL MODEL AND STUDY PARTICIPANT DETAILS

#### Animals

Mice (8–16 weeks old) of both sexes were used for the experiments. We considered these mice adult, as they were aged beyond the early critical period for cortical plasticity. C57BL6/J, *Nt5e*^*−/−*^ (JAX 018986), *ChAT*^*Cre*^ (JAX 006410), and *DAT*^*Cre*^ mice (JAX 006660) were purchased from the Jackson Laboratory (JAX). *Gfap*^*CreER*^ mice were a gift from Suzanne Baker’s lab.^97^ The care and use of animals were reviewed and approved by the Institutional Animal Care and Use Committee at St. Jude Children’s Research Hospital.

### METHOD DETAILS

#### Fast-scanning cyclic voltammetry *in vivo*

##### Fabrication of carbon fiber microelectrodes

Carbon fiber microelectrodes were constructed as previously described.^69^ In brief, a single 7-μm carbon fiber (Cytec Thornel, T300) was inserted into a short 10- to 15-mm borosilicate glass capillary (1.5 mm × 0.86 mm, Sutter Instruments) that had a microscopic tip pulled using a pipette puller (P-2000, Sutter Instruments). The tip with exposed carbon fiber was sealed during flame etching of the carbon fiber, as described previously.^99^ The exposed carbon fiber was then trimmed with a scalpel to a final length of 100–150 μm. Electrical connection with 10-mm long chlorided silver wire soldered to a 1-mm gold pin (Warner Instruments) was made by back-filling the capillary with a high ionic–strength solution (4 M potassium acetate, 150 mM potassium chloride). The connection between the glass capillary and the pin was sealed with epoxy glue. The silver–silver chloride (Ag/AgCl) reference electrode was fabricated by chloriding a silver wire. The electrodes were soaked in 2-propanol for at least 10 min prior to use.

##### Instrumentation

Fast-scanning cyclic voltammograms were collected using an 85004 Tethered FSCV Mouse System (Pinnacle Technology Inc.). Data-acquisition software and hardware were used to apply the triangular waveform and collect the resultant current data through an acquisition box. For detection of adenosine, the electrode was scanned from −0.4 to 1.5 V and back at 400 V/s every 100 ms. The reference electrode was a Ag/AgCl electrode.

##### Calibration of carbon fiber microelectrodes

Microelectrodes were calibrated using a custom-built, in-house flow-injection system. The carbon fiber microelectrode was positioned at the output of a flow-injection apparatus. The buffer was gravity-fed at 2 mL/min flow rate. Compounds were injected for 3–5 s to mimic fast concentration changes that occur in the brain. To stabilize the background current, electrodes were cycled using the experimental waveform (−0.4–1.5 V, 400 V/s, 10 Hz) for 15 min before collecting any cyclic voltammograms. Carbon fiber electrodes were tested by injecting adenosine, ATP, ADP, AMP, inosine, guanine, guanosine, dopamine, acetylcholine, or adenine at different concentrations (0.1, 0.5, 1, 2, 5 μM). Electric current versus time traces were plotted, and peak current was measured for each compound. Cyclic voltammograms were background-subtracted by averaging 10 background scans taken directly before the compound was injected into the flow chamber. For each electrode, 3 replicates were collected and averaged for each data point.

##### Surgery and implantation of the carbon fiber microelectrode

Mice were anesthetized with isoflurane (induction 2%, maintenance 1.0%–1.5% in pure oxygen), and an ~1-mm hole was drilled into the mouse skull over the ACx. The carbon fiber microelectrode was implanted into the ACx (~300–400 μm below the pial surface). Another hole was drilled in the skull over the contralateral cortex for the reference electrode (chloridized Ag wire). Both electrodes were secured with dental cement. The animal was given meloxicam (5 mg/kg), Baytril (10 mg/kg), and dexamethasone (2 mg/kg) immediately after surgery and for 3 days to reduce postoperative pain and prevent postoperative infection and swelling. After surgery, the mice recovered on a warm pad for 2 h. Most experiments were performed in awake, freely moving mice. Some experiments were performed in mice anesthetized with isoflurane (2.0% induction, 1.0%–1.5% maintenance in pure oxygen) or sodium pentobarbital (50 mg/kg induction, 20 mg/kg maintenance).

##### Sound stimulation

Broadband noise (white noise, bandwidth 0–50 kHz, 70-dB SPL) or pure tones with frequencies ranging 4.8–29.4 kHz, attenuations 0–40 dB, and durations 100 ms to 5 s played in pseudo-random order. All acoustic stimuli were generated by a free-field electrostatic speaker placed 10 cm from the contralateral ear of the mouse by using the OpenEx software and an RZ6 signal processor with 100-MHz processor speed (TDT).

In some experiments, SEAR was measured by FSCV in response to pairing acoustic stimuli with tail shock in freely moving mice. After establishing the SEAR baseline, 5 rounds of electrical stimulation, delivered to the animal’s tail via an ISO-Flex stimulus isolator driven by the Master-8 pulse generator (AMPI), were paired with 5 broadband noises (5-s duration). Each electrical stimulation (50 pulses at 100 Hz, pulse 0.5 ms, 0.5–2 mA) preceded the acoustic stimulus by 300–400 ms. When the tail shock was unpaired with acoustic stimulation, electric stimulation preceded the acoustic stimulus by 20 s.

#### *In vivo* two-photon adenosine imaging

##### Surgery

Mice were anesthetized with 2% isoflurane (in pure oxygen), and under aseptic conditions, a midline incision was made in the scalp. *AAV5-hSyn:GRAB*_*Ado-1M*_ was injected into the left MGv (300 nL at a rate of 10 nL/min; 3.2 mm caudal to bregma, 2 mm lateral to midline, with an injection depth of 2.8 mm). After viral injection, the left temporalis muscle was excised. The scalp was closed by suturing, and the animal was allowed to recover on a warm pad for 24 h. The animal was injected with meloxicam (2 mg/kg per day for as many as 3 days) to reduce postoperative pain and stress. Postoperative recovery was monitored for 3 days. Four weeks later, under isoflurane anesthesia and aseptic conditions, a headpost was attached to the skull with miniature stainless-steel screws and dental cement, and craniotomy and duratomy were performed over the left ACx. A dental cement well was placed around the craniotomy, and a 3-mm glass coverslip was cemented over the craniotomy. Dental cement was also used to seal the ipsilateral ear of the mouse. The animal was given meloxicam (2 mg/kg) to reduce postoperative pain and allowed to recover on a warm pad for 4–12 h after surgery.

##### Imaging GRAB_Ado_ fluorescence activity in awake mice

After recovery from surgery, the awake mouse was stabilized on the two-photon microscope stage via its headpost and allowed to walk along a rotating disc. To determine the sound-responsive area in the ACx, we monitored GRAB_Ado_ fluorescence of axon terminals from the MGv. Imaging was performed approximately 300–400 μm beneath the pial surface during exposure to bursts of broadband noise (white noise, bandwidth 0–50 kHz, 70-dB SPL, 5-s duration). Two-photon imaging was performed using an Olympus FVMPE-RS multiphoton laser-scanning microscope, a tunable femtosecond-pulsed laser unit (InSight, Spectra-Physics, 930-nm excitation), a water immersion 25× objective (NA 1.05, Olympus XLPLN25XWMP2) at sampling speed 0.067 (ms/pixel), and a resonant scanner. Tones were generated by a free-field electrostatic speaker placed 10 cm from the contralateral ear of the mouse by using the OpenEx software and an RZ6 signal processor (100 MHz) (TDT). In some experiments, GRAB_Ado_ imaging was performed in mice anesthetized with ketamine (induction, IP: ketamine 100 mg/kg and xylazine 10 mg/kg; maintenance ketamine 50 mg/kg body weight) or sodium pentobarbital (induction lP: 50 mg/kg; maintenance 20 mg/kg).

##### Measuring GRAB_Ado_ fluorescence and FSCV in acute slices and cultured neurons

Mice injected with *AAV5-hSyn:GRAB*_*Ado-1M*_ were anesthetized and acute 400-μm brain slices containing portions of the MGv and ACx were cut and maintained in a perfused bath as previously described.^100,101^ FSCV and two-photon imaging of *GRAB*_*Ado*_ were performed in the same set of slices in response to different concentrations of exogenous adenosine. Imaging analysis in cultured neurons was performed as previously described.^71^

#### *In vivo* calcium imaging

##### Surgery

Injections of virus and installation of the headpost and cortical window were carried out using similar protocols described above for *in vivo* adenosine experiments. *AAV5-hSyn-jGCaMP8f* was injected into the left ACx (300 nL at a rate of 25 nL/min; 1.9 and 2.3 mm caudal to bregma, 0.3 mm medial to the dorsal insertion of the temporalis muscle, with an injection depth of 0.8 mm). Initial calcium-imaging experiments evoked plasticity in the ACx by pairing electrical stimulation of the NB with a pure-tone presentation. In these experiments, a custom concentric electrode (26-gauge, part F11041, P Technologies) was implanted 0.5-mm caudal to bregma, 1.5 mm lateral to the midline, and at a depth of 4.5 mm beneath the brain surface.

Later plasticity experiments paired optogenetic stimulation of either the NB or VTA axons in the ACx with a tone presentation to evoke ACx plasticity. For optogenetic stimulation specific to the NB, 250 nL of *AAV-Ef1a-DIO-hChR2(E123T/T159C)-EYFP* was injected (25 nL/min) into *ChAT*^*Cre*^ mice at the left NB coordinates 0.5 mm caudal to bregma, 1.5 mm left lateral to midline, at a depth of 4.5 mm beneath the brain surface. For optogenetic stimulation specific to the VTA, 250 nL *AAV-Ef1a-DIO-hChR2(E123T/T159C)-EYFP* was injected (25 nL/min) into *DAT*^*Cre*^ mice at left VTA coordinates 3.25 mm caudal to bregma, 0.5 mm left lateral to midline, at a depth of 4.5 mm beneath the brain surface. Lastly, *AAV5-CaMKIIa:Nt5e-GFP (AAV5-CaMKIIa:GFP-2A-Nt5e*) was injected into the left MGv as a means of overexpressing *Nt5e* in excitatory TC neurons. *AAV-CaMKIIa:GFP* was injected into the left MGv as the control (see below). Correct positioning of the stimulus electrodes and virus injection sites were verified for all mice via histologic assessment.

##### Measuring cortical plasticity in the ACx of awake mice: two-photon imaging

After recovery from surgery, the awake mouse was stabilized via its headpost on the two-photon microscope stage and allowed to walk along a rotating disc. Within the ACx, we monitored jGCaMP8f fluorescence 300–400 μm beneath the pial surface, which corresponds to L3/4 thalamorecipient neurons, during exposure to pure tones, with frequencies ranging from 4.8-29.4 kHz, intensities of 15- to 75-dB SPL (60-0 dB SPL attenuation, respectively), and a duration of 50 ms played at 1 Hz in pseudo-random order. Two-photon imaging was performed using Ultima IV (Prairie Technologies) and the Ti:Sapphire laser Ultra II (Coherent). Tones were generated by a free-field electrostatic speaker placed 10 cm from the contralateral ear and using the OpenEx software and an RZ6 signal processor (100 MHz) (TDT). Each frequency-attenuation combination was delivered 75 times during the 30-min imaging session for a total of 1800 tone pulses. Neurons within the 710 × 710-μm field of view were imaged using a water immersion 25× objective (NA 1.05, Olympus XPlan N) and scanned at an excitation wavelength of 920 nm, at a rate of 10 frames/s using a resonant scanner. Background noise in the vicinity of the mouse was reduced by placing a sound attenuation chamber around the mouse and objective. During scanning, the resonant scanner on the microscope emitted a continuous 7.9 ± 0.015 kHz frequency measuring approximately 50 dB SPL within the sound attenuation chamber. Other background noise frequencies were below 1.5 kHz at 30–35 dB SPL. These sources of noise did not interfere with our analyses because they did not overlap with the frequencies or timing of pure tones used in this study.

GCaMP6f labeling via viral expression or transgene expression techniques typically gives neurons a donut-like appearance, which indicates that the GCaMP is expressed in the cytoplasm but not the nucleus. However, this donut-like appearance in neurons expressing jGCaMP8f is often very faint and difficult to discern due to such low baseline fluorescence (Figure 4B). Also, it is rare to find neurons that overexpress jGCaMP8f and show calcium-signal saturation, which could indicate an unhealthy cell. When such a saturated cell is detected, it is rejected from the data analysis.

Video sequences were motion stabilized to align each frame to a selected reference frame and jGCaMP8f fluorescence signals were extracted from the stabilized video sequence as previously described.^102^ First, active neurons were identified manually in a max-projection image of all frames of the image sequence. Second, a narrow region of interest (ROI) surrounding each soma was created, and the total fluorescence intensities of the ROIs were calculated for each frame of the sequence to yield a jGCaMP8f activity value at each time point.

Activity peaks were automatically detected by finding all local maxima in the postprocessed signal that exceeded a user-defined threshold and had rise and decay kinetics like those of jGCaMP8f. Activity signals were normalized to baseline levels, and peak amplitudes were calculated as (ΔF/F) × 100%, where F is the baseline fluorescence intensity within the ROI of the selected neuron. The percentage of neurons within a sampling region responsive to any tone widely ranges from about 8% to more than 30%; the average is ~18%.

##### Imaging analysis

After the evoked activity peaks were identified, they were assigned to the corresponding sound-stimuli parameters (frequency, attenuation, and timing). Average fluorescence values were calculated for each cell at every frequency/attenuation combination as a function of time before, during, and after sound stimulation (Figure 4C). Peak activity values that were not within 200 ms after the sound stimuli were determined to be non–sound related and were automatically excluded from analyses. These values were used to identify the TF of each neuron as previously described^55^

Two methods were used to represent and analyze changes in TFs after tone training. TFs for all sound-responsive neurons, before and after tone training within a given treatment, were compared by creating cumulative percentile distributions that displayed the accumulated percentage of neurons analyzed on the ordinate and the TFs on the abscissa. The Kolmogorov-Smirnov (K-S) test was used to determine whether there was a significant difference in the pre-vs. post-training distributions. Second, to better illustrate the presence or absence of a shift in the TF of neurons after tone training, we created heat maps (ΔTF maps) depicting the TF measured pre-training on the ordinate and that measured post-training on the abscissa. The color intensity of the heatmap (0%–100%) indicated the percentage of neurons that began with TF (ordinate) for pre-training measurements and ended with TF (abscissa) for post-training measurements. If there was no change in the TF of any neuron for a given treatment, the resultant heatmap would show 100% color intensity along the diagonal (slope = 1). Neurons with a TF (ordinate) above 9.8 kHz that shifted toward 9.8 kHz post-training would show increased color intensity above the diagonal. Conversely, neurons with a TF (ordinate) below 9.8 kHz that shifted toward 9.8 kHz post-training would show increased color intensity below the diagonal.

To test for significant deviations from a slope of 1, we hypothesized that the presentation of a 9.8-kHz tone combined with NB or VTA stimulation would shift the TF values away from their initial value and toward the training frequency. The absolute (ABS) difference between the neuron’s TF and the training frequency (9.8 kHz), before and after tone training, was calculated as follows: [ΔTF = ABS(TF_before_ − 9.8 kHz) − ABS(TF_after_ − 9.8 kHz)]. If there was no change in the TF with tone training, ΔTF = 0. A positive value indicated a shift toward the training frequency, and a negative value indicated a shift away from it. The Wilcoxon signed-rank test was performed on the values, comparing ΔTFs before and after tone training, for all sound-responsive neurons and for all sets of experimental and control treatments.

#### Induction of cortical plasticity in vivo: pairing NB or VTA electrical or optogenetic stimulation with acoustic stimuli

Mice were anesthetized with isoflurane, and then their heads were fixed in a stereotaxic device (David Kopf Instruments). For electrical stimulation, an incision was made in the scalp, a small hole was drilled for the craniotomy under aseptic conditions, and a concentric bipolar electrode (cat. no. F11041, Plastic One) was implanted into the NB or VTA, immediately prior to implanting either the carbon microelectrode for FSCV experiments or the window for imaging experiments. The tip of the concentric bipolar electrode was positioned either in the left NB (coordinates from the bregma: anterior–posterior 0.5 mm, medial–lateral 1.5 mm, dorsal-ventral 4.5 mm) or left VTA (anterior–posterior 3.25 mm, medial–lateral 0.5 mm, dorsal-ventral 4.5 mm). After several days of recovery from surgery, we conducted FSCV or imaging. In FSCV experiments, a 5-s broadband (white) noise was delivered every 60 s to evoke SEAR. After establishing a SEAR baseline, 5 rounds (at 0.016 Hz) of electrical stimulations of the NB or VTA were paired with the broadband noise. Each round of electrical stimulation was introduced 100–200 ms prior to the sound and consisted of 100 pulses (0.5-ms duration, 150–300 mA) at 100 Hz. In some FSCV-pairing experiments, the delay between electrical stimulation and onset of the acoustic stimuli ranged from −10 s to 10 s.

In optogenetic experiments, mice were anesthetized with isoflurane and immobilized in a stereotaxic device. *AAV-Ef1a-DIO-hChR2(E123T/T159C)-EYFP* was injected into the NB of *ChAT*^*Cre*^ mice or into the VTA of *DAT*^*Cre*^ mice. Viruses were injected via a 33-gauge metal cannula (Plastics One). In FSCV experiments, 3–4 weeks after the viral injections, a carbon fiber microelectrode and an optic fiber (Prizmatix, fiberoptic cannulae ferrule 1.25 mm, fiber core 250 μm, NA 0.66) were implanted. An incision was made in the scalp, and 3 holes were drilled for the carbon fiber electrode, the Ag/AgCl reference electrode, and the optic fiber; 2-3 additional holes were drilled for the anchor screws. The following stereotaxic coordinates were used for implantations (from the bregma): 1) carbon fiber microelectrode in the left ACx (anterior–posterior, −2 mm; medial–lateral, −0.3 from the temporal muscle; dorsal-ventral, −0.8 mm); 2) optic fiber in the NB (anterior–posterior, −0.5 mm; medial–lateral, +1.5 mm; dorsal-ventral, −4.3 mm); 3) optic fiber in the VTA (anterior–posterior, −3.25 mm; medial–lateral, +0.5 mm; dorsal-ventral, −4.3 mm). The Ag/AgCl reference electrode was placed 1- to 2-mm deep into the contralateral cortex. In behavioral experiments, *AAV-Ef1a-DIO-hChR2(E123T/T159C)-EYFP* was injected bilaterally into the NB of *ChAT*^*Cre*^ mice or into the VTA of *DAT*^*Cre*^ mice. Three to 4 weeks later, the optic fibers were implanted bilaterally (anterior–posterior, −2 mm; lateral ± 0.3 mm from the edge of the temporal muscle); on the surface of the cortical area) in ChR2-expressing *ChAT*^*Cre*^ or *DAT*^*Cre*^ mice to stimulate NB or VTA projections to the ACx. Following implantation, electrodes, fibers, and screws were secured with dental cement. Mice were placed on a heating pad for recovery for ~2 h. Mice were given Baytril (10 mg/kg), dexamethasone (2 mg/kg), and meloxicam (5 mg/kg) for 3 days to prevent infections and inflammation and to reduce pain. Experiments were performed several days after surgery. During experiments, 5 rounds at 0.016 Hz of the optical stimulation were delivered using the Prizmatix Optogenetics-LED (Prizmatix Ltd). Each round consisted of a train (1 s at 80 Hz) of blue light pulses (8 ms) delivered 100–200 ms prior to the sound stimulation. In two-photon calcium-imaging experiments, pure tones and electrical or optogenetic stimulation of the NB or VTA were paired in a similar fashion.

#### Electrical stimulation

After imaging ACx neuronal responses to sound stimuli for 30 min (pretraining TF mapping), a series of electrical stimuli paired with pure tones was presented to the mouse as a means of evoking ACx cortical plasticity. Specifically, a 1-s train of current pulses (400–600 mA, 100 Hz, 0.5-ms pulse width) was delivered to the NB via an implanted concentric bipolar electrode. A 9.8-kHz pure tone (75 dB, 100-ms duration) was then delivered 500 ms after the NB stimulus began. This pairing was repeated 10 times at 0.033 Hz. The 30-min post-training TF mapping resumed 10 min thereafter, as previously described for pre-pairing.

#### Optogenetic stimulation

*ChAT*^*Cre*^ mice with ChR2-expressing neurons in the NB (see above) were used. A 1-s train of 470-nm LED pulses was delivered to the imaging ROI through the two-photon objective (pulse frequency, 80 Hz; pulse width, 8 ms, 7.5 mW (LED 4D067/LED driver DC4104, Thorlabs). We delivered 10 of these pairings at 0.033 Hz, and 10 min thereafter, we proceeded to another 30 min of post-pairing TF mapping. Lastly, we repeated the plasticity experiments with optogenetic stimulation of the VTA in *DAT*^*Cre*^ mice expressing ChR2 in the VTA (see above).

#### Auditory fear conditioning

To test the role of adenosine in traditional auditory associative fear learning, we modified a protocol (Figure 6A) from^78^ and used mice that had received a viral injection to cause overexpression of NT5E or GFP. In brief, mice were placed in a quiet pre-testing room for 2 h/day for 2 days. On the third day, baseline frequency-discrimination acuity at 16.4 kHz was tested in each mouse in a separate room, as described below. On the fourth day, we ran auditory fear conditioning (AFC) experiments using Med Associates equipment (MED-VFC-USB-R). In brief, mice learned to differentiate between 2 frequencies in a sound-attenuating chamber. Video Freeze software (ver. 2.7.3.0) was used for data collection and analyses.

First, mice learned to differentiate between 16.4 kHz (CS+) and 8.2 kHz (CS−). This protocol consisted of a 3-min chamber acclimation period, followed by the presentation of 16 alternating CS+ and CS− auditory cues (20.5-s duration each, 80-db SPL, 2-min interstimulus interval), paired with a co-terminating mild shock (0.5 s, 0.5 mV, CS+) or not paired with a shock (CS−). Animals were then moved to a quiet room for 2 h before being returned to the animal holding room. After 2 days off, mice learned to differentiate between 16.4 kHz (CS+) and 13.94 kHz (CS−) in a similar fashion. The next day, mice were moved to a quiet room for 2 h and then tested for frequency-discrimination acuity at 16.4 kHz (post-AFC).

#### TC LTP and TC LTD

Long-term synaptic plasticity experiments were performed in acute TC slices containing the ACx and portions of the MGv, as previously described.^66,67^

##### TC slice preparation

Brains were placed in cold (4°C) dissecting artificial cerebrospinal fluid (ACSF) containing 125 mM choline-Cl, 2.5 mM KCl, 0.4 mM CaCl_2_, 6 mM MgCl_2_, 1.25 mM NaH_2_PO_4_, 26 mM NaHCO_3_, and 20 mM glucose (300–310 mOsm), with 95% O_2_/5% CO_2_. TC slices were obtained from the left hemisphere by using a slicing angle of 15°. After a 1-h incubation in ACSF [125 mM NaCl, 2.5 mM KCl, 2 mM CaCl2, 2 mM MgCl_2_ (1 mM MgCl_2_ in pairing experiments), 1.25 mM NaH_2_PO_4_, 26 mM NaHCO_3_, and 20 mM glucose (300–310 mOsm), with 95% O_2_/5% CO_2_]at room temperature, the slices were transferred into the recording chamber and superfused (2–3 mL/min) with warm (30°C–32°C) ACSF.

##### Whole-cell electrophysiology

Whole-cell recordings were obtained from cell bodies of L3/4 thalamorecipient neurons in the ACx. Patch pipettes (open pipette resistance, 3.5–5 MΩ) were filled with an internal solution containing 125 mM CsMeSO_3_, 2 mM CsCl, 10 mM HEPES, 0.1 mM EGTA, 4 mM MgATP, 0.3 mM NaGTP, 10 mM Na_2_ creatine phosphate, 5 mM QX-314, 5 mM tetraethylammonium (TEA) Cl, and 10–25 μM Alexa Fluor 594 (pH 7.4 was adjusted with CsOH, 290–295 mOsm). QX-314 was included to block the generation of action potentials in recorded neurons. Alexa Fluor 594 was added for visualization of dendritic structures during all experiments. Only neurons with dendritic spines (indicative of excitatory neurons) and a well-established primary apical dendrite extending to the pial surface (indicative of pyramidal neurons) were chosen. Neurons with any sign of dendritic damage were excluded from the analysis. Voltage-clamp recordings were made using a Multiclamp 700B (Molecular Devices), digitized (10 kHz; DigiData 1440A; Molecular Devices), and recorded using pClamp 10.0 software (Molecular Devices). Excitatory postsynaptic currents (EPSCs) were recorded at holding membrane potentials of −70 mV. In all experiments, membrane potentials were corrected for a liquid junction potential of −10 mV. TC EPSCs were evoked by current pulses (100-μs duration) delivered to the thalamic radiation via tungsten bipolar electrodes (FHC, Inc.) placed in the white matter, midway between the MGv and the ACx (rostral to the hippocampus). Stimulation intensities (50–300 μA) were adjusted during each experiment to elicit 150–200 pA EPSCs. Recordings were discarded if access resistance was >25 MΩ or if access resistance changed >20% during the course of the whole-cell recording.

##### TC LTP and LTD experiments

TC LTP was induced by electrical stimulation of the thalamic radiation by using a high-frequency train (40 Hz) of square electrical pulses (100 μs). Picrotoxin (5 mM) was added to the intracellular solution.^67^ The 40-Hz LTP-induction protocol consisted of 3 periods of stimulation delivered every 5 min. Every period consisted of 10 trains of 40-Hz stimulations delivered for 200 ms every 5 s. In all LTP experiments, baseline EPSCs were recorded using test stimulations in voltage-clamp mode (holding potential, −70 mV) for up to 3 min before induction of LTP. LTP was induced in current-clamp mode while maintaining a steady resting potential of −70 mV using slow (t = 5 s) current injection. After induction, the recordings were resumed in voltage-clamp mode at the same preinduction stimulation rate.

TC LTD was induced by a low-frequency train (900 pulses at 1 Hz) of square electrical pulses (100 μs) delivered to the thalamic radiation. Baseline EPSCs were recorded using test stimulations in voltage-clamp mode (holding potential, −70 mV) for up to 5 min before induction of LTD. LTD was induced in current-clamp mode, while maintaining a steady resting potential of −70 mV by using slow (t = 5 s) current injection in the presence of carbachol (5 μM).^66^ After induction, the recordings were resumed in voltage-clamp mode at the same preinduction stimulation rate. TC LTP and LTD experiments were analyzed offline. We compared EPSCs (initial 2-ms slope) recorded 30–70 min after LTP induction or 60–75 min after LTD induction; baseline EPSCs were recorded before induction of either LTP or LTD.

#### Overexpression of NT5E in the auditory thalamus *in vivo*

Mice were anesthetized with isoflurane and their heads were then fixed in a stereotaxic device. An incision was made in the scalp, and small holes were drilled for the craniotomy. *AAV5-CaMKIIα:Nt5e-GFP (AAV5-CaMKIIa:GFP-2A-Nt5e*) or control *AAV-CaMKIIα:GFP* (250 nL) were injected via a 33-gauge metal cannula (Plastics One) into the MGv. The following coordinates from the bregma were used: anterior–posterior, −3.2 mm; medial–lateral, ±2.0 mm; dorsal-ventral, −2.8 mm). In behavioral experiments, we injected the viruses bilaterally, and in FSCV or imaging experiments, we injected them ipsilaterally. In some experiments, viral injections into the MGv were combined with viral injections into the NB or VTA (see above). Mice were allowed to recover for 2–3 h on a heated pad; several weeks later, they were used for FCSV, two-photon imaging, or behavioral experiments.

#### Auditory perceptual plasticity

Auditory perceptual plasticity, in the form of changes in experience-dependent frequency-discrimination acuity, was measured using the PPI of ASR.^77^ The ASR and PPI of ASR were measured using a computer interface (SM1000-II, Kinder Scientific) and methods modified from Clause et al.^77^ In brief, a 70-db SPL background tone (16.4 kHz) was played throughout the session, unless otherwise noted. Each session included the following 4 blocks: Block 1 was a 5-min acclimation period, during which the background tone was played. Block 2 included startle-only trials, in which a 120-db SPL, 20-ms broadband (white) noise burst was played. Block 3 contained the prepulse trials and 10 startle-only trials in a pseudo-random order. Each prepulse trial consisted of a prepulse (a pure tone 0%, 1%, 2%, 4%, 8%, 16%, or 32% lower than the background) played for 80 ms at 70-db SPL followed by the 120-db SPL, 20-ms broadband noise startle pulse, returning to the background tone after the startle. Every pre-pulse frequency in Block 3 was presented 10 times. Block 4 included startle-only trials to examine any habituation that occurred during the session. The intertrial interval was 10–20 s, and the startle magnitude was the maximum force exerted immediately after the startle pulse. All trials were recorded as .wav files that were created using Audacity 2.1.2 software, to ensure smooth transitions from pure tone to pure tone and from pure tone to broadband noise startle.

PPI was calculated as (1 − [prepulse trial startle/startle-only trial]) × 100. Values for each animal then had a logistic-regression curve fitted to the normalized PPI percentages at each prepulse frequency to determine the frequency at which 50% of the total PPI was achieved, subsequently called the FDT; animals with an *r*^*2*^ < 0.7 were excluded from further analyses. FDT values were analyzed using a *t* test, a 1-way ANOVA, or a paired *t* test, as appropriate. Pure-tone frequencies and sound intensities were calibrated daily by using the sound-level meters NL-52 (Rion Co., LTD) and SMSPL Rev B (Kinder Scientific), respectively.

In pairing experiments, we tested pre-pairing auditory acuity and then 1 day later, we paired (10 times at 0.016 Hz) pure tones (16.4 kHz or 9.8 kHz, 50 ms) with electrical (250 ms, 100 Hz) or optogenetic (1 s, 80 Hz) stimulation of the NB or VTA (see above). During pairing, the pure tone onset coincided with the middle of an electrical stimulation of the NB or VTA cell bodies or optical stimulation of the NB or VTA projections in the ACx. One to 3 h after the pairing protocol, we re-tested the auditory acuity in the same animals. The change in FDT after pairing was the measure of auditory perceptual plasticity.

#### Generation of *Nt5e*–conditional knockout mice

A CRISPR/Cas9 approach was used to generate mice with the *Nt5e*-floxed allele. Two sgRNAs were designed to target the *Nt5e* introns 1 and 2. Two single-stranded oligos, each containing a *loxP* site and two 70- to 90-bp homology arms, were used as the DNA donors. The following oligonucleotides were used for cloning sgRNA T7 expression vectors (System Biosciences): Nt5esg1: 5'-AGG GCT CAC TGG GAA GAG GAA ACG-3', 5'- AAA CCG TTT CCT CTT CCC AGT GAG-3'; and Nt5esg2: 5'-AGG GAG AGC ATC TAG GTC AAC TGG-3', 5'-AAA CCC AGT TGA CCT AGA TGC TCT-3'. *In vitro* transcription was performed using the MEGAshortscript T7 Kit (Thermo Fisher), and transcribed RNAs were purified using MEGAclear RNA Kit (Thermo Fisher). The following single-stranded oligo DNA donors were generated as Ultramer DNA oligos (Integrated DNA Technologies): Nt5e-F, 5'-GTG TTA GAA GAG GTG TAT GTG CAT TGC ACA AGC CAG GTT CAT TAC TAA ACT ATT TGT TGT GCT TAC TTA CCT GCC TCA CTG GGA AGA GGA ATT AAT AAC TTC GTA TAA TGT ATG CTA TAC GAA GTT ATA CGA GGT GGA GGA GAA TGT ACC ATG GCC CTT GTT AGC ATC AAG GAC CTA GAG CCA GTG CTG CCA GGC CTC TC-3'; Nt5e-R, 5'- CTT TAT GAG ACT TTT GCA TTT GGG ACA CTT TCA TTT TAG TGC CGG TTC TTA GAA ACT AAC CTG AGA GCA TCT AGG TCA ACA TAA CTT CGT ATA ATG TAT GCT ATA CGA AGT TAT TAA TGG AGG ACT GCC TTG CAA TAG AGT GCT GGG TGA GAC TTC AGG ACG CCA TGT CGG TGG TCC CCT CGT GAC CAC TGA AGA CTT GC-3'. A mixture of 50 ng/μL sgRNAs, 100 ng/μL hCas9 (System Biosciences), and 100 ng/μL ssODN (single-stranded oligodeoxyribonucleotide) donors were injected into the pronucleus of oocytes of C57BL/6J zygotes (St. Jude Protein Production Core). The injected oocytes were then returned to culture media (M16 or Advanced-KSOM, both from Millipore); later the same day, they were transferred to Day 0.5 pseudo-pregnant foster dams. Pups were born after 19 days’ gestation and sampled at postnatal days 7–10 for genotyping via targeted next-generation sequencing. The F0 mice were genotyped by PCR using primers Nt5eloxP1-F: 5'-AGTGCATTGTTTGGAGGGTGGTGT-3', Nt5eloxP1-R: 5'-ACTTCCTCGGTACCTAAATCCACTGA-3' and Nt5eloxP2-F: 5'-GTCGATGGCTGTGTCTCCCTTAGTCT-3', Nt5eloxP2-R: 5'-GCCCTGGGAATCCTATGCTGAGA-3'.

PCR products were purified and analyzed by agarose gel electrophoresis. The sequences of PCR products with correct band sizes were further confirmed by sub-cloning and Sanger sequencing. F0 mice with correct mutation sites were crossed with C57BL/6J mice. The genotype of F1 mice was confirmed by PCR analysis and Sanger sequencing (as described above) and then backcrossed to C57BL/6J for at least 5 generations to further eliminate potential off-target events. Resultant *Nt5e*^*fl/+*^ mice were crossed with mice expressing Cre (e.g., *Gfap*^*CreER*^ mice) or injected with AAVs expressing Cre. Experiments in adult *Gfap^CreER^;Nt5e^fl/fl^* mice were performed 4–6 weeks after tamoxifen treatment (100 mg/kg body weight, IP; T5648, Sigma) for 3 consecutive days. The tamoxifen solution was prepared by dissolving the agent in corn oil (C8267, Sigma) at a concentration of 20 mg/mL at 37°C. The solution was then filter-sterilized and stored at 4°C in the dark.

#### Quantitative PCR

RNA was isolated from the mouse thalamus by using the mirVana RNA isolation kit (Life Technologies). The SuperScript III reverse transcriptase kit (Life Technologies) was used to synthesize cDNA from 500 ng total RNA. Quantitative PCR (qPCR) was performed using SYBR Green master mix (Applied Biosystems) with the following primers (forward and reverse, respectively): *Gapdh*: 5'-GTCGGTGTGAACGGATTTG-3' and 5'-TAGACTCCACGACATACTCAGCA-3', *U6*: 5'-CGCTTCGGCAGCACATATAC-3' and 5'-TTCACGAATTTGCGTGTCAT-3', *Adora1*: 5'-TGTGCCCGGAAATGTACTGG-3' and 5'-TCTGTGGCCCAATGTTGATAAG-3', *Grin1*: 5'-CTGCGACCCCAAGATTGTCAA-3' and 5'-TATTGGCCTGGTTTACTGCCT-3', *Nt5e*: 5'-AACCCCTTTCCTCTCAAATCCA-3' and 5'-CAGGGCGATGATCTTATTCACAT-3'. Expression levels of *Nt5e, Adora1*, and *Grin1* mRNAs were normalized to the housekeeping genes *Gapdh* or *U6* for each sample. Samples from each mouse were run in triplicate. Expression was quantified using the standard curve method.

#### Western blotting

Mouse MGv tissues were lysed in ice-cold RIPA buffer [50 mM Tris-HCl (pH 7.4), 1% NP-40, 0.25% sodium deoxycholate, 150 mM NaCl, and 1 mM EDTA] that included protease inhibitor cocktail tablets. A total of 20 μg protein was loaded per lane. Sodium dodecyl sulfate/polyacrylamide gel electrophoresis, protein transfer to polyvinylidene difluoride membranes, and Western blotting were performed using standard methods. Primary antibodies used were rabbit anti-Nt5e (1:250, AP2014b; Abgent) and mouse anti–β-actin (1:10,000, A5316; Sigma-Aldrich). Secondary antibodies used were anti-rabbit (1:30,000, 926–68021; LI-COR Biotechnology) and anti-mouse (1:15,000, 926–32212; LI-COR) antibodies conjugated to infrared dye 680 or 800. Blots were imaged and quantified using the Odyssey infrared imaging system (LI-COR).

### QUANTIFICATION AND STATISTICAL ANALYSIS

Data are presented as means ± SEM. All statistics and statistical methods can be found in the figure legends and the main text. Statistics were computed using the SigmaPlot (Systat Software, Inc.) or Prism (GraphPad, Dotmatics) software. Differences in mean data were considered significant if the p value of the test result was less than 0.05.

## Supplementary Material

1

## Figures and Tables

**Table:** KEY RESOURCES TABLE

REAGENT or RESOURCE	SOURCE	IDENTIFIER
Antibodies		
Rabbit anti-Nt5e	Abgent	Cat# AP2014b; RRID: AB_2236185
Mouse anti–β-actin	Sigma-Aldrich	Cat# A5316; RRID: AB_476743
IRDye 680LT goat anti-rabbit IgG	LI-COR Biotechnology	Cat# 926-68021; RRID: AB_10706309
IRDye 800CW donkey anti-mouse IgG	LI-COR Biotechnology	Cat# 926-32212; RRID: AB_621847
Recombinant Viruses		
*AAV5-CaMKIIα:Cre-GFP*	This paper	N/A
*AAV5-CaMKIIa:eGFP*	This paper	N/A
*AAV5-hSyn:GRAB_Ado-1M_*	This paper	N/A
*AAV5-Ef1α-DIO-hChR2(E123T/T159C)-EYFP*	Addgene	Cat# 35509-AAV5
*AAV5-hSyn-jGCaMP8f*	This paper	N/A
*AAV5-CaMKIIα:Nt5e-GFP (AAV5-CaMKIIa:GFP-2A-Nt5e)*	This paper	N/A
Chemicals, peptides, and recombinant proteins		
Adenosine	Tocris	Cat# 3624
ATP	Sigma-Aldrich	Cat# A7699
ADP	Tocris	Cat# 3633
AMP	Sigma-Aldrich	Cat# A1752
Inosine	Sigma-Aldrich	Cat# I4125
Guanine	Sigma-Aldrich	Cat# G11950
Guanosine	Sigma-Aldrich	Cat# G6752
Dopamine	Tocris	Cat# 3548
Acetylcholine	Tocris	Cat# 2809
Adenine	Sigma-Aldrich	Cat# A8626
Experimental models: Organisms/strains		
*ChAT*^*Cre*^ mice	Jackson Laboratory	JAX 006410
*DAT*^*Cre*^ mice	Jackson Laboratory	JAX 006660
*Gfap*^*CreER*^ mice	(Chow et al.)^97^	N/A
*Nt5e*^*−/−*^ mice	Jackson Laboratory	JAX 018986
*Nt5e*^*fl/fl*^ mice	This paper	N/A
Oligonucleotides		
See the list in methods.		
Recombinant DNA		
*pGP-AAV-syn-jGCaMP8f*	Zhang et al.^98^	Addgene plasmid # 162376
*pENN.AAV.CaMKII.HI.GFP-Cre.WPRE.SV40*	a gift from James M. Wilson	Addgene plasmid # 105551
*pAAV-CaMKIIa-eGFP*	a gift from Bryan Roth	Addgene plasmid # 50469
Software and algorithms		
pCLAMP 10.0	Molecular Devices	N/A
Prism	GraphPad	N/A
SigmaPlot	Systat	N/A
ImageJ	NIH	https://imagej.nih.gov/ij/
FSCV 2.0.9 software	Pinnacle Technology, Inc	N/A

## Highlights

- Acoustic stimuli cause adenosine release in the auditory cortex (ACx) within seconds
- Sound-evoked adenosine release (SEAR) originates from thalamocortical projections
- Cholinergic and dopaminergic inputs or mild shock deter SEAR via synaptic mechanisms
- Associative ACx plasticity and auditory learning occur in adults if SEAR is inhibited
