# Supplementary material for: Sound-evoked adenosine release in cooperation with neuromodulatory circuits permits auditory cortical plasticity and perceptual learning
Source: Cell Rep. Author manuscript; Available in PMC 2024 Mar 14. (PMC10939737; doi:10.1016/j.celrep.2024.113758)
Supplement: 1 [file NIHMS1970890-supplement-1.pdf]

Cell Reports, Volume *43*

## Supplemental information

# Sound-evoked adenosine release in cooperation with neuromodulatory circuits permits auditory cortical plasticity and perceptual learning

**Ildar T. Bayazitov, Brett J.W. Teubner, Feng Feng, Zhaofa Wu, Yulong Li, Jay A. Blundon, and Stanislav S. Zakharenko**

## Supplemental Information

## Supplemental Figures

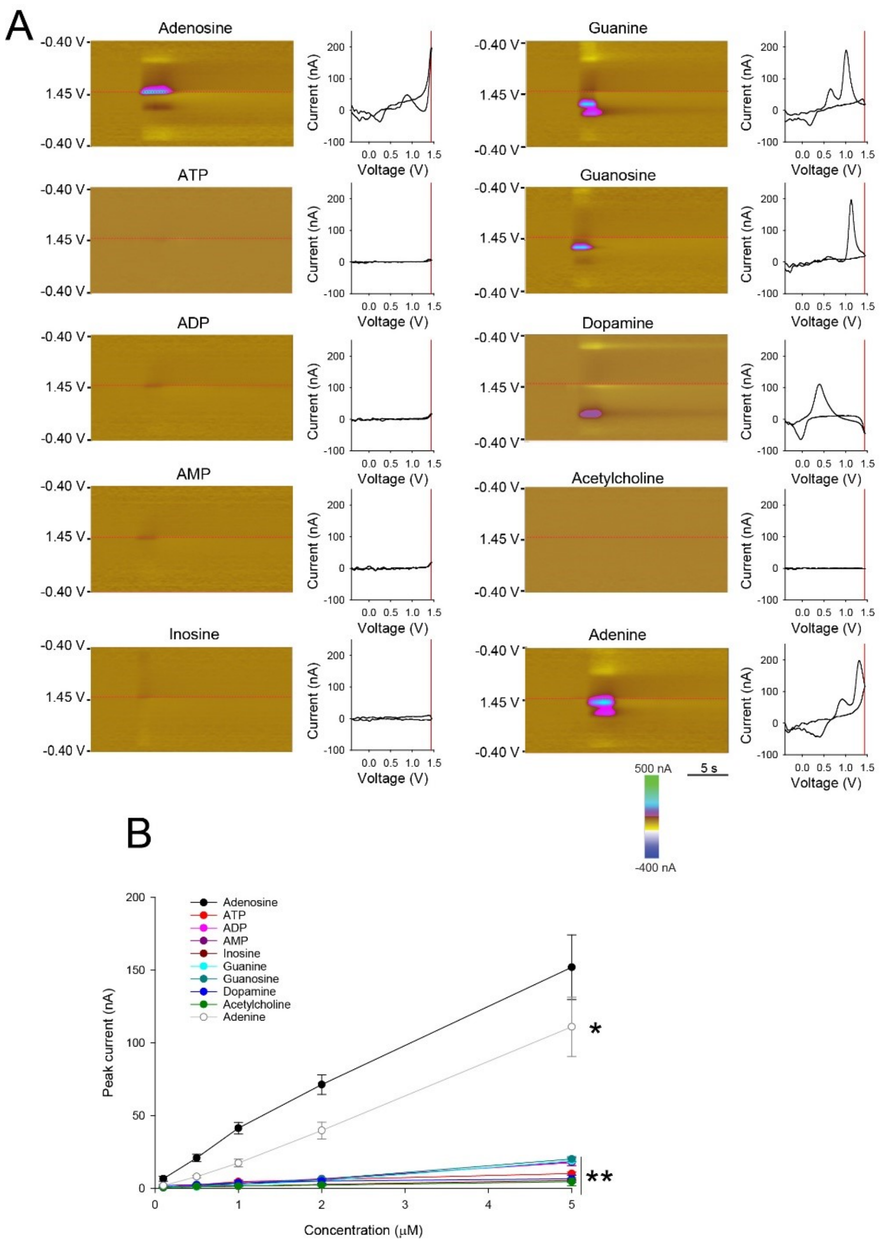


**Figure S1**. **Specificity of adenosine detection by fast-scanning cyclic voltammetry (related to Figure 1).**

(**A**) Examples of false color plots (left panels) and cyclic voltammograms (right panels) in response to bath application of adenosine, ATP, ADP, AMP, inosine, guanine, guanosine, dopamine, acetylcholine, or adenine at different concentrations in vitro. Red lines mark the primary adenosine oxidation at 1.4 V.

(**B**) Mean oxidation peak (measured at 1.4 V) as a function of concentrations of adenosine, ATP, ADP, AMP, inosine, guanine, guanosine, dopamine, acetylcholine, or adenine (2-way RM ANOVA, $F_{9,4} = 27.22$, ***p* <0.001; post-hoc Holm-Sidak test: adenosine vs. ATP, ADP, AMP, inosine, guanine, guanosine, dopamine, or acetylcholine ***p* <0.001, adenosine vs. adenine **p* = 0.012). All bath applications were done in triplicate.

Averaged data are presented as the mean ± SEM.

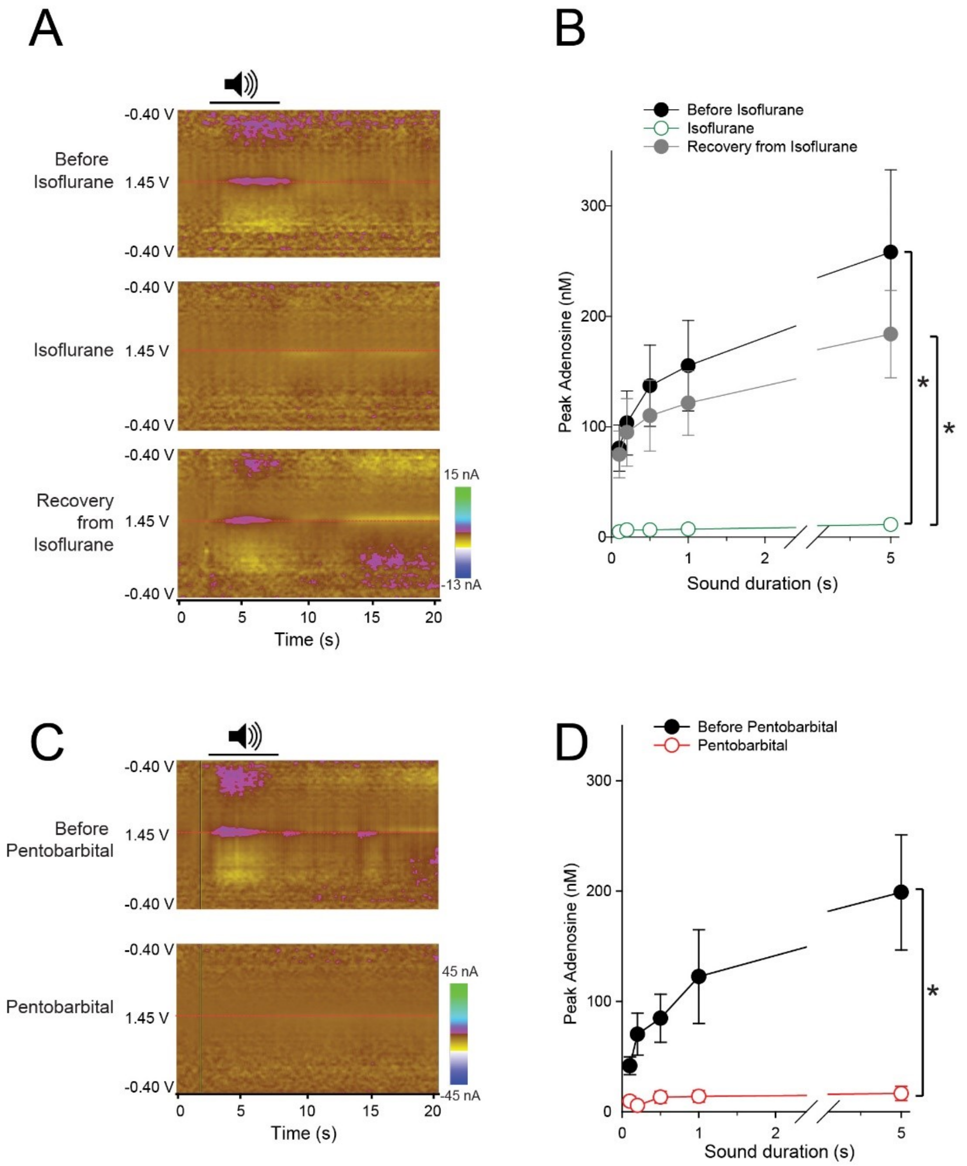


**Figure S2**. **Anesthesia eliminates SEAR in the ACx, as measured by fast-scanning cyclic voltammetry (related to Figure 1).**

(**A, B**) Examples of false color plots (**A**) and the mean peak in SEAR in the ACx, as a function of broadband noise duration (**B**) before, during, and after recovery from isoflurane (1.5% in pure oxygen) anesthesia (2-way RM ANOVA, $F_{2,4}$ = 5.1 *$p$ <0.001, treatment *$p$ = 0.003, 6-10 mice).

(**C, D**) Examples of false color plots (**C**) and mean peak SEAR in the ACx, as a function of broadband noise duration (**D**) before and during anesthesia with sodium pentobarbital (20 mg/kg) (2-way RM ANOVA, $F_{1,4}$ = 7.3 $p$ <0.001, treatment *$p$ <0.01, n = 3 mice).

Averaged data are presented as the mean ± SEM.

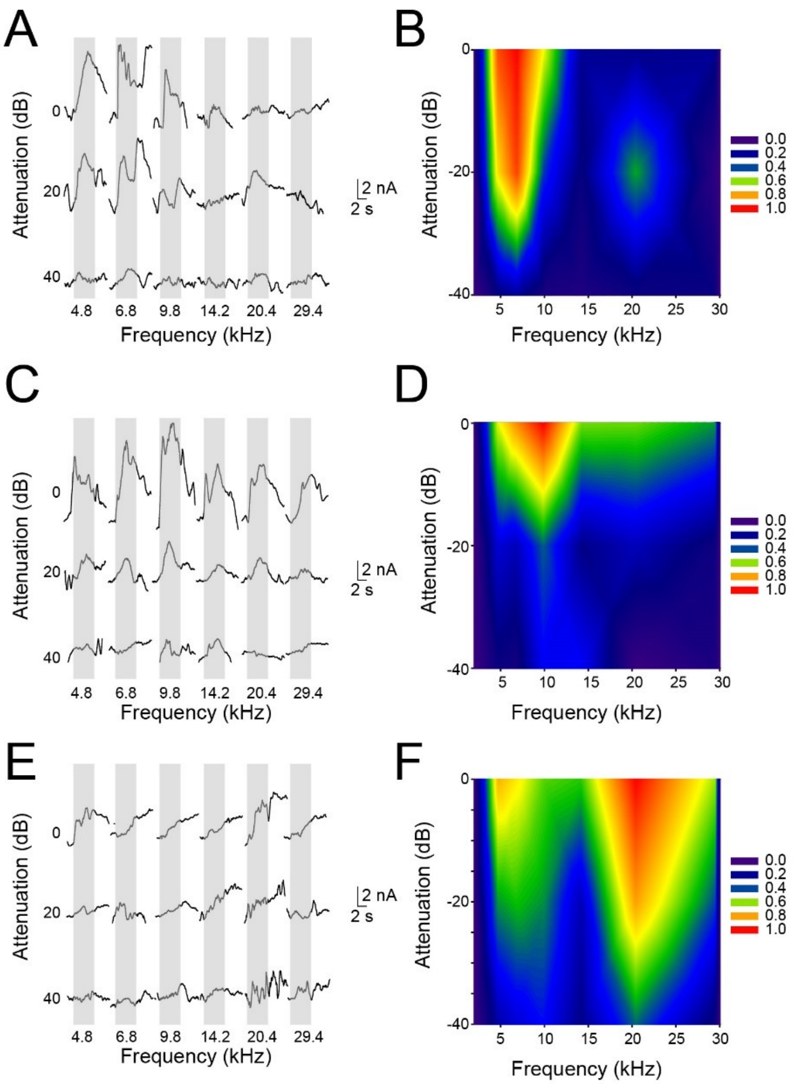


**Figure S3**. **SEAR in the ACx in response to pure tones (related to Figure 1).**
Three examples of sound-evoked responses (**A**, **C**, **E**) and respective receptive fields (**B**, **D**, **F**) measured by fast-scanning cyclic voltammetry in response to pure tones of different frequencies (4.8-29.4 kHz) and different intensities (0-, 20-, 40-db SPL attenuations). Gray bars denote sound presentation.

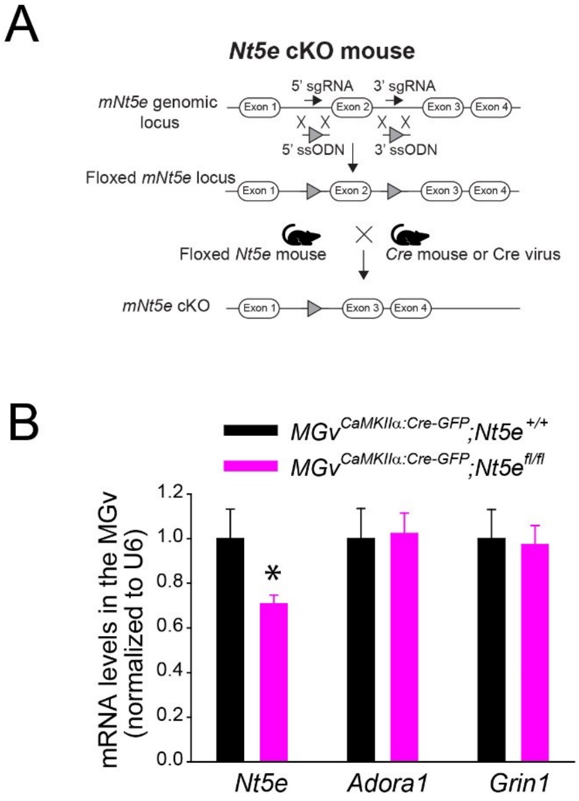


**Figure S4**. **Production and validation of mutant mice with conditional deletion of *Nt5e* (related to Figure 1).**

(**A**) Schematic for engineering *Nt5e*–conditional knockout (cKO) mice. The sgRNAs were designed to target the 5'- and 3'-ends of exon 2 of the *Nt5e* gene. A single-stranded oligodeoxyribonucleotide (ssODN) consisting of homology arms flanking a LoxP site (arrowhead) was designed to insert the LoxP site directly into each corresponding sgRNA cut site (2 ssODNs total). Floxed *Nt5e* animals were either crossed with $Gfap^{CreER}$ mice or injected with AAVs encoding Cre to generate the conditional deletion of *Nt5e* in different populations of cells.

(**B**) *Nt5e* was reduced in the auditory thalamus (MGv) of $Nt5e^{fl/fl}$ mice injected with *AAV-CaMKIIa:Cre-GFP* into the MGv ($MGv^{CaMKIIa:Cre-GFP;}Nt5e^{fl/fl}$ mice), as determined by qPCR analysis of the levels of *Nt5e, Adora1*, and *Grin1* expressed in $MGv^{CaMKIIa:Cre-GFP;}Nt5e^{fl/fl}$ mice (n = 7) and WT controls injected with *AAV-CaMKIIa:Cre-GFP* into the MGv ($MGv^{CaMKIIa:Cre-GFP;}Nt5e^{+/+}$ mice, n = 8). Mann-Whitney rank-sum test: *Nt5e*: $U$ = 8, $^*p$ = 0.02; *Adora1*: $U$ = 19, $p$ >0.05. *Grin1*: unpaired 2-tailed $t$-test $t_{13}$ = 0.165, $p$ >0.05.
Averaged data are presented as the mean ± SEM.

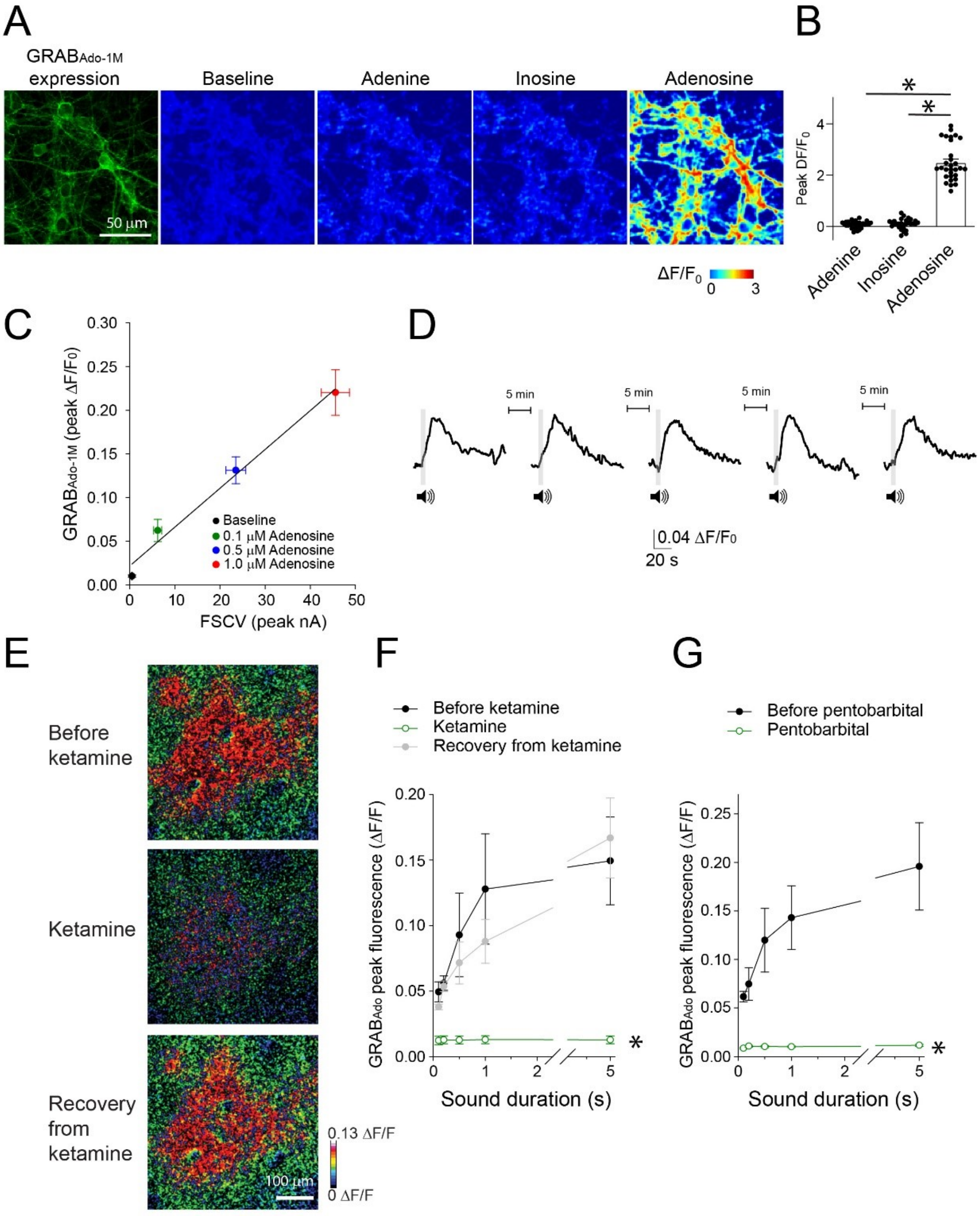


**Figure S5**. **SEAR in the ACx, as measured by the adenosine-specific fluorescent indicator GRAB$_{Ado}$, is eliminated by anesthesia (related to Figure 1).**

(**A**) Images of GRAB$_{Ado-1M}$ expression and responses to adenine (20 μM), inosine (20 μM), and adenosine (20 μM) in rat hippocampal neurons in culture (10–15 days in vitro). Scale bar, 50 μm.

(**B**) Quantification of peak fluorescence changes after the application of adenine (n = 30), inosine (n = 30), or adenosine (n = 30) to rat hippocampal neuronal culture expressing GRAB$_{Ado-1M}$ (Kruskal-Wallis 1-way ANOVA on ranks, $H_2 = 59.6$, *$p$ <0.001; post-hoc Tukey test: adenosine vs. adenine *$p$ <0.001; adenosine vs. inosine *$p$ <0.001).

(**C**) The correlation between FSCV and $GRAB_{Ado-1M}$ responses to different concentrations of adenosine in acute ACx brain slices ($r^2$ = 0.984; *p* = 0.008, n = 5).
(**D**) $GRAB_{Ado-1M}$ responses in the ACx to repeated acoustic stimulations (white noise, 5 s, grey bars).
(**E**) Images of SEAR in the ACx (calculated as the difference between $GRAB_{Ado}$ fluorescence evoked by broadband noise and baseline $GRAB_{Ado}$ fluorescence) before, during, and after anesthesia with ketamine/xylazine. Scale bar, 100 μm.
(**F, G**) Average peak SEAR as a function of broadband noise duration before, during, and after recovery from ketamine anesthesia (**F**: 2-way RM ANOVA, $F_{2,4}$ = 3.5, **p* = 0.008, treatment **p* = 0.011, n = 3 mice) or before and during sodium pentobarbital (20 mg/kg)–induced anesthesia (**G**: 2-way RM ANOVA, $F_{1,4}$ = 5.4 **p* = 0.005, treatment **p* = 0.008, n = 3 mice).
Averaged data are presented as the mean ± SEM.

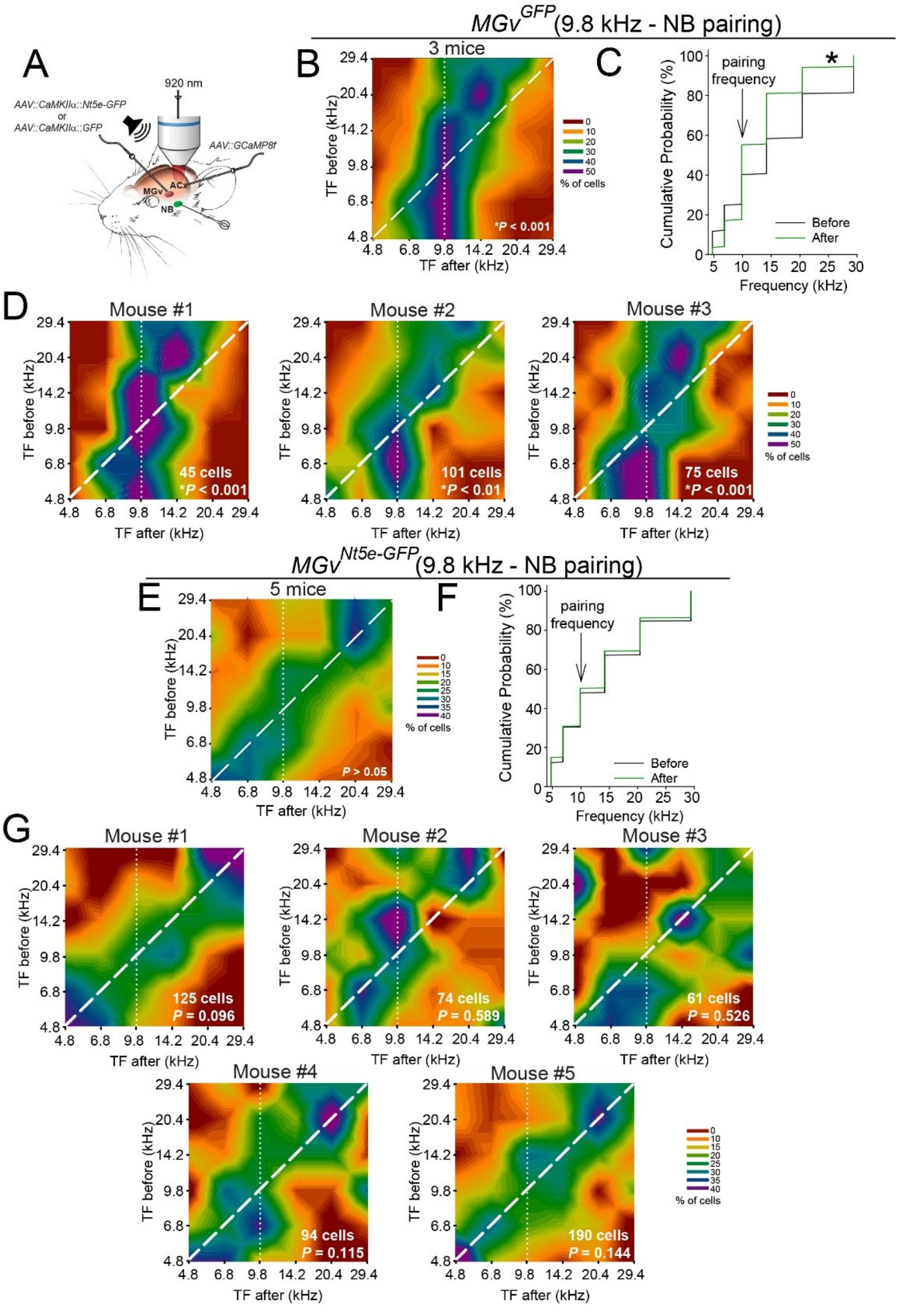


**Figure S6. Cortical plasticity induced by pairing a sound with electrical stimulation of the NB is eliminated by thalamic overexpression of NT5E (related to Figure 4).** (**A**) Schematic showing 2-photon microscopic (920 nm) imaging of GCaMP8f in the ACx of *MGv*$^{Nt5e\text{-}GFP}$ mice or *MGv*$^{GFP}$ mice exposed to pure tones of different frequencies or intensities (speaker), while the left NB is locally stimulated by a bipolar electrode.

(**B, C**) Heat map (ΔTF map) depicting shifts in neuron numbers (normalized to percent sampled) (**B**) and cumulative histogram of TFs of recorded neurons in the ACx (**C**) combined for 3 mice, before and after pairing a pure tone (9.8-kHz) with electrical stimulation of the NB in WT $MGv^{GFP}$ mice, 221 neurons, 3 mice, Wilcoxon signed-rank test $Z = -7.5$, $*p < 0.001$ (**B**), K-S test $D = 0.22$, $*p < 0.001$ (**C**).
(**D**) Heat maps (ΔTF maps) depicting shifts in neuron numbers (normalized to percent sampled) in each mouse in (**B**). Data for each mouse (1 site per mouse) are shown. The numbers of cells and *p*-values (Wilcoxon signed-rank test) for each mouse are shown in the bottom right corners.
(**E, F**) Heat maps (ΔTF maps) depicting shifts in neuron numbers (normalized to percent sampled) (**E**) and cumulative histograms of TFs of recorded neurons in the ACx (**F**) combined for 5 mice before and after pairing a pure tone (9.8-kHz) with electrical stimulation of the NB in $MGv^{Nt5e\text{-}GFP}$ mice, 544 neurons, 5 mice, Wilcoxon signed-rank test $Z = -0.84$, $p = 0.400$ (**E**), K-S test $D = 0.03$, $p = 0.993$ (**F**).
(**G**) Heat maps (ΔTF maps) depicting shifts in neuron numbers (normalized to percent sampled) in each mouse in (**E**). Data for each mouse (one site per mouse) are shown. The numbers of cells and the *p*-values (Wilcoxon signed-rank test) for each mouse are shown in the bottom right corners.
In heat maps, diagonal lines represent no change in TFs after pairing, and vertical lines represent 9.8 kHz (pairing frequency).

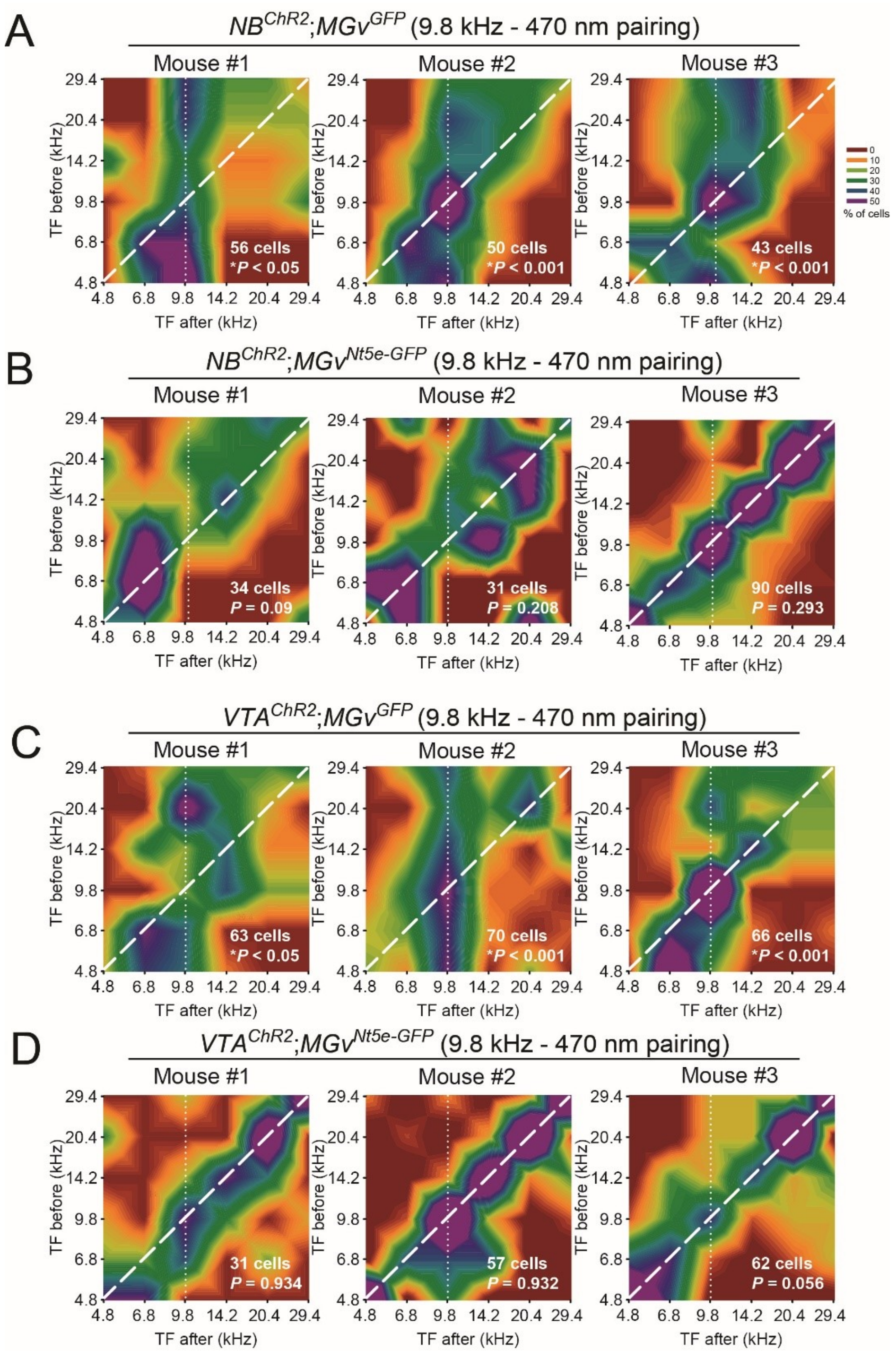


**Figure S7**. **Cortical plasticity induced by pairing sound with optogenetic activation of the NB or VTA and measured in individual mice with or without thalamic overexpression of NT5E (related to Figure 4).**

(**A, B**) Heat maps (ΔTF maps) depicting shifts in neuron numbers (normalized to percent sampled) before and after pairing a pure tone (9.8-kHz) with optogenetic stimulation (470 nm) of the NB in WT $NB^{ChR2}$;$MGv^{GFP}$ mice (**A**) or $NB^{ChR2}$;$MGv^{Nt5e\text{-}GFP}$ mice (**B**). (**C, D**) Heat maps depicting shifts in neuron numbers (normalized to percent sampled) before and

after pairing a pure tone (9.8-kHz) with optogenetic stimulation of the VTA in WT *VTA*$^{ChR2}$*;MGv*$^{GFP}$ mice (**C**) or *VTA*$^{ChR2}$*;MGv*$^{Nt5e\text{-}GFP}$ mice (**D**). Data for each mouse (one site per mouse) in every group are shown. The numbers of cells and *p*-values (Wilcoxon signed-rank test) for each mouse are shown in the bottom right corners. Diagonal lines represent no change in TFs after pairing, and vertical lines represent 9.8 kHz (pairing frequency).

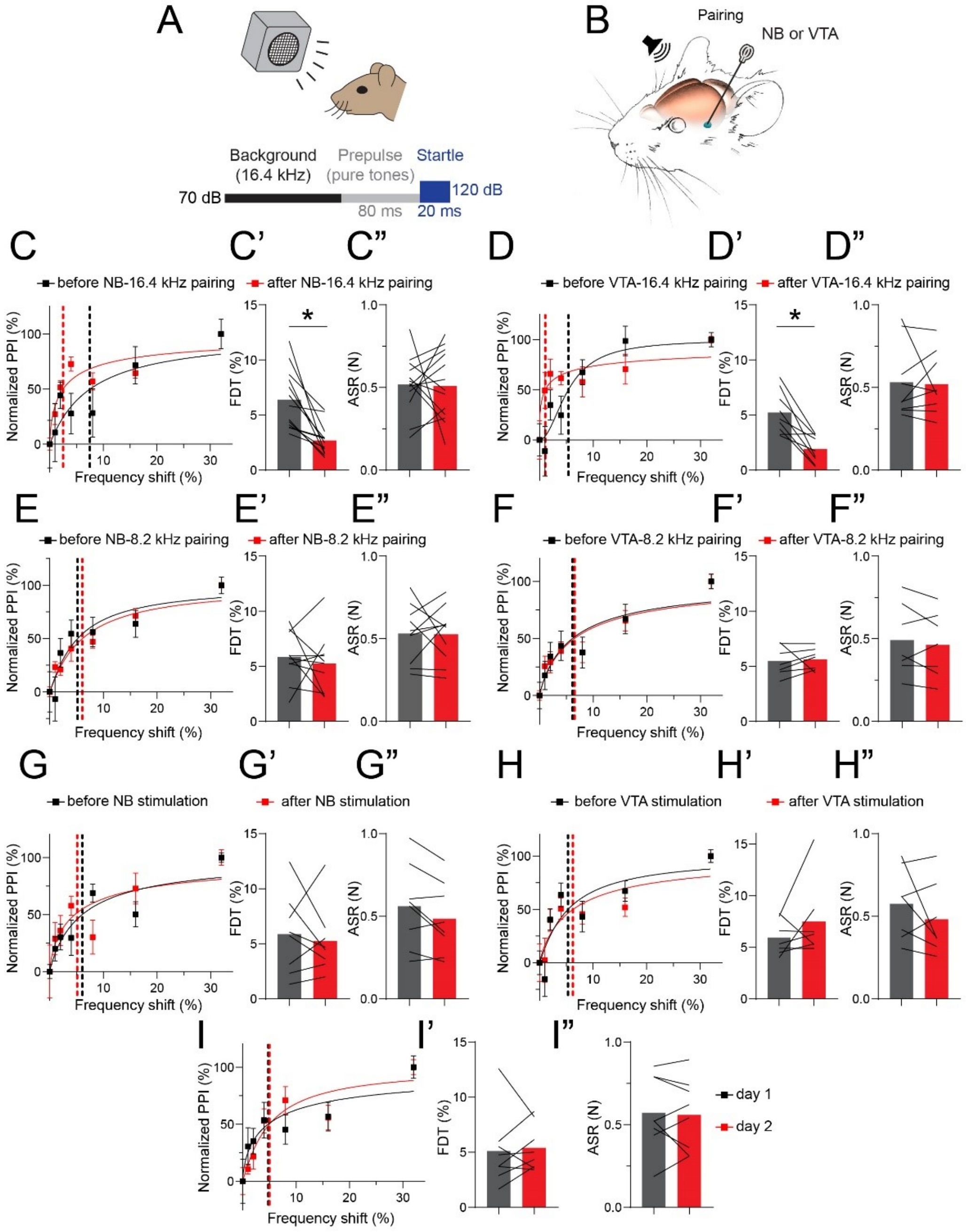


**Figure S8**. **Frequency-specific perceptual plasticity induced by pairing a pure tone with stimulation of the NB or VTA (related to Figure 5).**

(**A**) Schematic of the behavioral protocol for testing frequency-discrimination acuity. A background tone (16.4 kHz, 70-dB SPL) is present during the entire experiment. Variable frequency pre-pulse tones (80 ms, 70-dB SPL) are presented prior to the startle stimulus (white noise, 20 ms, 120-dB SPL).

(**B**) Schematic showing the NB–or VTA–sound pairing procedures. Electrical stimulation of the left NB or VTA was paired with a pure tone (16.4 kHz or 8.2 kHz, 70-dB SPL).

(**C-C"**) Example of recordings from a mouse of normalized PPI magnitude, as a function of frequency difference between background and pre-pulse tones (**C**), before and after NB–16.4 kHz pairing. Points and error bars are mean ± SEM of 10 repeated measurements in the same animal. Solid lines are logistic-regression fits to these points; dotted lines are frequency-discrimination thresholds (FDTs). (**C'**) The FDT is smaller after NB–16.4 kHz pairing than it was before. Two-tailed paired *t*-test, $t_{12}$ = 4.7, **p* = 0.0005 (n = 13 mice). (**C"**) The acoustic startle response (ASR) does not change after NB–16.4 kHz pairing. Two-tailed paired *t*-test, $t_{12}$ = 0.12, *p* = 0.91 (n = 13 mice).
(**D-D"**) Example of recordings of normalized PPI magnitude, as a function of frequency difference between background and pre-pulse tones (**D**), FDT, 2-tailed paired *t*-test, $t_8$ = 4.96, **p* = 0.0011 (n = 9 mice) (**D'**), and ASR, 2-tailed paired *t*-test, $t_8$ = 0.179, *p* = 0.862 (n = 9 mice) (**D"**) before and after VTA–16.4 kHz pairing.
(**E-E"**) Example of recordings of normalized PPI magnitude, as a function of frequency difference between background and pre-pulse tones (**E**), FDT, 2-tailed paired *t*-test, $t_9$ = 0.553, *p* = 0.593 (n = 10 mice) (**E'**), and ASR, 2-tailed paired *t*-test, $t_9$ = 0.088, *p* = 0.932 (n = 10 mice) (**E"**), before and after NB–8.2 kHz pairing.
(**F-F"**) Example of recordings of normalized PPI magnitude as a function of frequency difference between background and pre-pulse tones (**F**), FDT, 2-tailed paired *t*-test, $t_6$ = 0.302, *p* = 0.773 (n = 7 mice) (**F'**), and ASR, 2-tailed paired *t*-test, $t_6$ = 0.904, *p* = 0.4 (n = 7 mice) (**F"**) before and after VTA–8.2 kHz pairing.
(**G-G"**) Example of recordings of normalized PPI magnitude as a function of frequency difference between background and pre-pulse tones (**G**), FDT, 2-tailed paired *t*-test, $t_7$ = 0.498, *p* = 0.633 (n = 8 mice) (**G'**), and ASR, 2-tailed paired *t*-test, $t_7$ = 2.25, *p* = 0.06 (n = 8 mice) (**G"**) before and after NB stimulation alone.
(**H-H"**) Example of recordings of normalized PPI magnitude as a function of frequency difference between background and pre-pulse tones (**H**), FDT, 2-tailed paired *t*-test, $t_6$ = 1.18, *p* = 0.281 (n = 7 mice) (**H'**), and ASR, 2-tailed paired *t*-test, $t_6$ = 1.14, *p* = 0.298 (n = 7 mice) (**H"**) before and after VTA stimulation alone.
(**I-I"**) Example of recordings of normalized PPI magnitude as a function of frequency difference between background and pre-pulse tones (**I**), FDT, 2-tailed paired *t*-test, $t_7$ = 0.33, *p* = 0.751 (n = 8 mice) (**I'**), and ASR, 2-tailed paired *t*-test, $t_7$ = 0.283, *p* = 0.785 (n = 8 mice) (**I"**) on Days 1 and 2.
Averaged data are presented as the mean ± SEM.

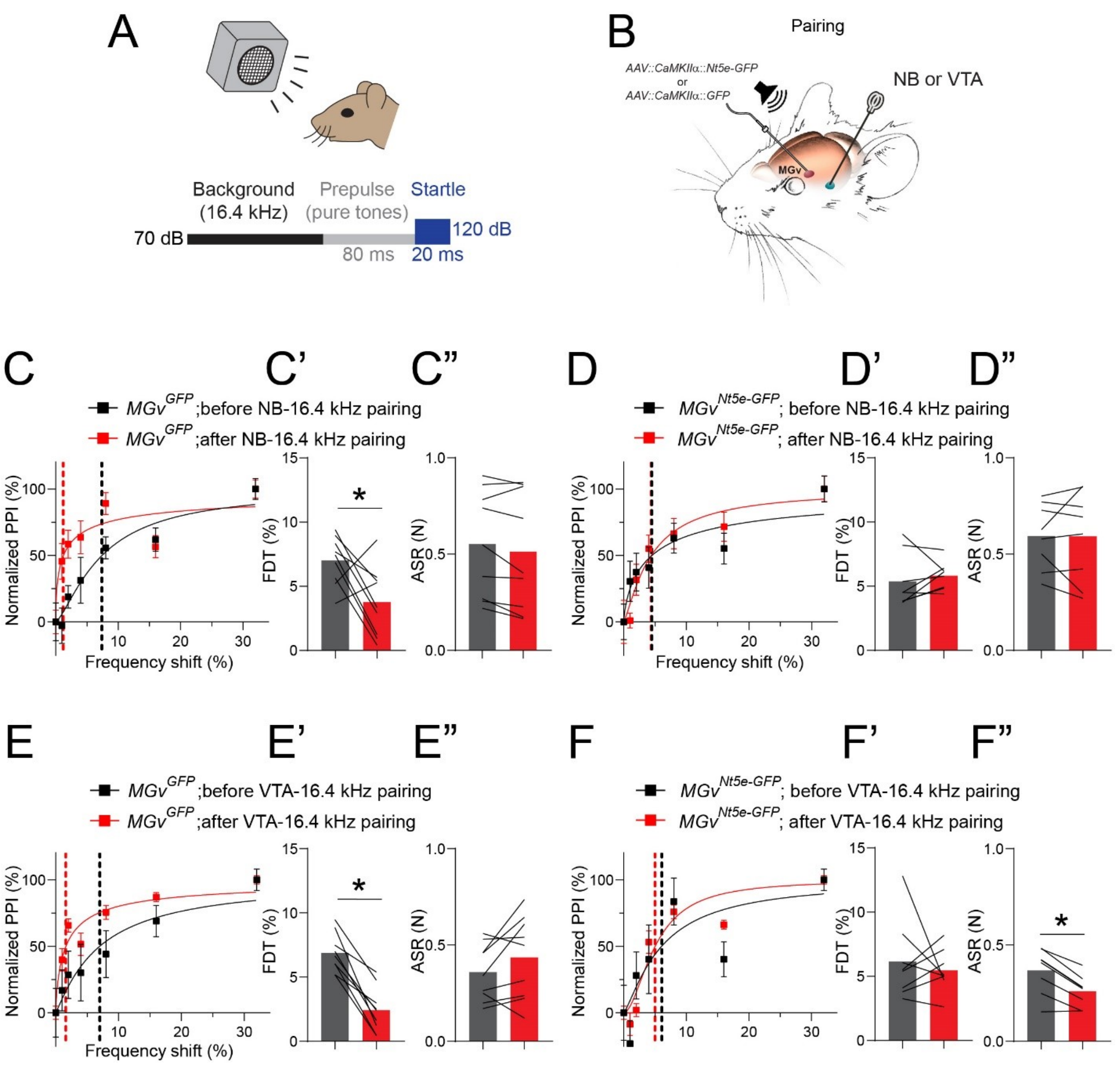


**Figure S9**. **Overexpression of NT5E in the auditory thalamus (MGv) prevents associative frequency-specific perceptual plasticity induced by pairing a pure tone with stimulation of the NB or VTA (related to Figure 5).**

(**A**) Schematic of the behavioral protocol for testing frequency-discrimination acuity (as in **Fig. S7A**).

(**B**) Schematic showing the NB–sound or VTA–sound pairing procedures in $MGv^{Nt5e\text{-}GFP}$ or $MGv^{GFP}$ mice. Electrical stimulation of the left NB or VTA was paired with a pure tone (16.4 kHz, 70-dB SPL) in mice injected with *AAV-CaMKIIa-Nt5e-GFP* or *AAV-CaMKIIa-Nt5e-GFP* into the ipsilateral MGv.

(**C-C"**) Cortical plasticity induced by NB–16.4 kHz pairing in $MGv^{GFP}$ mice. Example of recordings from a mouse of normalized PPI magnitude, as a function of frequency difference between background and pre-pulse tones, before and after NB–16.4 kHz pairing in $MGv^{GFP}$ mice. Points and error bars are mean ± SEM of 10 repeated

measurements in the same animal. Solid lines are logistic-regression fits to these points; dotted lines are frequency-discrimination thresholds (FDTs). (**C'**) The FDT is smaller after NB–16.4 kHz pairing in *MGv*$^{GFP}$ mice than it was before. Two-tailed paired *t*-test, $t_8 = 2.74$, *$p = 0.025$ (n = 9 mice). (**C"**) The acoustic startle response (ASR) does not change after NB–16.4 kHz pairing in *MGv*$^{GFP}$ mice. Two-tailed paired *t*-test, $t_8 = 0.193$, $p = 0.09$ (n = 9 mice).

(**D-D"**) Overexpression of *Nt5e* in the MGv blocks the improved auditory perception induced by NB–16.4 kHz pairing. Example of recordings of normalized PPI magnitude as a function of frequency difference between background and pre-pulse tones (**D**), FDT, 2-tailed paired *t*-test, $t_7 = 0.748$, $p = 0.479$ (n = 8 mice) (**D'**), and ASR, 2-tailed paired *t*-test, $t_7 = 0.055$, $p = 0.956$ (n = 8 mice) (**D"**), before and after NB–16.4 kHz pairing in *MGv*$^{Nt5e\text{-}GFP}$ mice.

(**E-E"**) The improved auditory perception induced by VTA–16.4 kHz pairing in *MGv*$^{GFP}$ mice. Example of recordings of normalized PPI magnitude as a function of frequency difference between background and pre-pulse tones (**E**), FDT, 2-tailed paired *t*-test, $t_8 = 7.72$, *$p < 0.0001$ (n = 9 mice) (**E'**), and ASR, 2-tailed paired *t*-test, $t_8 = 1.655$, $p = 0.137$ (n = 8 mice) (**E"**), before and after VTA–16.4 kHz pairing in *MGv*$^{GFP}$ mice.

(**F-F"**) Overexpression of NT5E in the MGv blocks the improved auditory perception induced by VTA–16.4 kHz pairing. Example of recordings of normalized PPI magnitude as a function of frequency difference between background and pre-pulse tones (**F**), FDT, 2-tailed paired *t*-test, $t_7 = 0.543$, $p = 0.603$ (n = 8 mice) (**F'**), and ASR, 2-tailed paired *t*-test, $t_7 = 5.81$, *$p = 0.0007$ (n = 8 mice) (**F"**), before and after VTA–16.4 kHz pairing in *MGv*$^{Nt5e\text{-}GFP}$ mice.

Averaged data are presented as the mean ± SEM.
